# Biomarker Identification, Safety, and Efficacy of High-Dose Antioxidants for Adrenomyeloneuropathy: a Phase II Pilot Study

**DOI:** 10.1007/s13311-019-00735-2

**Published:** 2019-05-10

**Authors:** Carlos Casasnovas, Montserrat Ruiz, Agatha Schlüter, Alba Naudí, Stéphane Fourcade, Misericordia Veciana, Sara Castañer, Antonia Albertí, Nuria Bargalló, Maria Johnson, Gerald V. Raymond, Ali Fatemi, Ann B. Moser, Francesc Villarroya, Manuel Portero-Otín, Rafael Artuch, Reinald Pamplona, Aurora Pujol

**Affiliations:** 1 Neuromuscular Unit, Neurology Department, Bellvitge University Hospital, Feixa Llarga s/n, 08908 L’Hospitalet de Llobregat, Barcelona Spain; 2 Neurometabolic Diseases Laboratory, Bellvitge Biomedical Research Institute, Hospital Duran i Reynals, Gran Via de l’Hospitalet 199, 08908 L’Hospitalet de Llobregat, Barcelona Spain; 3 Center for Biomedical Research on Rare Diseases, Institute of Health Carlos III, Monforte de Lemos 3-5, Pabellón 11, 28029 Madrid, Spain; 4 Institute of Neuropathology, Bellvitge Biomedical Research Institute, Gran Via de l’Hospitalet 199, 08908 L’Hospitalet de Llobregat, Barcelona Spain; 5 Biomedical Research Institute of Lleida, Montserrat Roig 2, 25008 Lleida, Spain; 6 Neurophysiology Unit, Neurology Department, Hospital Universitari de Bellvitge, Feixa Llarga s/n, 08908 L’Hospitalet de Llobregat, Barcelona Spain; 7 Centre Bellvitge, Institut de Diagnòstic per la Imatge, Feixa Llarga s/n, 08908 L’Hospitalet de Llobregat, Barcelona Spain; 8 Department of Neuroradiology, Hospital Clínic, Barcelona, Spain; 9 Magnetic Resonance Imaging Core Facility, Institut D’Investigacions Biomèdiques August Pi i Sunyer (IDIBAPS), Barcelona, Spain; 10 Deparment of Neurology and Pediatrics, University of Minnesota Medical Center, 516 Delaware Street Southeast, Minneapolis, Minnesota 55455 USA; 11 Kennedy Krieger Institute, 707 North Broadway, Baltimore, Maryland 21205 USA; 12 Departament de Bioquimica i Biologia Molecular and Institute of Biomedicine of the University of Barcelona, Facultat de Biologia, Universitat de Barcelona, Avinguda Diagonal 645, 08028 Barcelona, Spain; 13 Center for Biomedical Research in Physiopathology of Obesity and Nutrition, Bellvitge Biomedical Research Institute, Monforte de Lemos 3-5, Pabellón 11, 28029 Madrid, Spain; 14 Institut de Recerca Sant Joan de Déu, Passeig de Sant Joan de Déu 2, 08950 Esplugues de Llobregat, Barcelona Spain; 15 Catalan Institution of Research and Advanced Studies, Passeig de Lluís Companys 23, 08010 Barcelona, Spain

**Keywords:** Adrenomyeloneuropathy, antioxidants, biomarkers, oxidative stress, inflammation.

## Abstract

X-Adrenoleukodystrophy (X-ALD) and its adult-onset, most prevalent variant adrenomyeloneuropathy (AMN) are caused by mutations in the peroxisomal transporter of the very long-chain fatty acid ABCD1. AMN patients classically present spastic paraparesis that can progress over decades, and a satisfactory treatment is currently lacking. Oxidative stress is an early culprit in X-ALD pathogenesis. A combination of antioxidants halts the clinical progression and axonal damage in a murine model of AMN, providing a strong rationale for clinical translation. In this phase II pilot, open-label study, 13 subjects with AMN were administered a high dose of α-tocopherol, N-acetylcysteine, and α-lipoic acid in combination. The primary outcome was the validation of a set of biomarkers for monitoring the biological effects of this and future treatments. Functional clinical scales, the 6-minute walk test (6MWT), electrophysiological studies, and cerebral MRI served as secondary outcomes. Most biomarkers of oxidative damage and inflammation were normalized upon treatment, indicating an interlinked redox and inflammatory homeostasis. Two of the inflammatory markers, MCP1 and 15-HETE, were predictive of the response to treatment. We also observed a significant decrease in central motor conduction time, together with an improvement or stabilization of the 6MWT in 8/10 subjects. This study provides a series of biomarkers that are useful to monitor redox and pro-inflammatory target engagement in future trials, together with candidate biomarkers that may serve for patient stratification and disease progression, which merit replication in future clinical trials. Moreover, the clinical results suggest a positive signal for extending these studies to phase III randomized, placebo-controlled, longer-term trials with the actual identified dose. ClinicalTrials.gov Identifier: NCT01495260

**Electronic supplementary material:**

The online version of this article (10.1007/s13311-019-00735-2) contains supplementary material, which is available to authorized users.

## Introduction

X-Adrenoleukodystrophy (X-ALD) is the most frequently inherited leukodystrophy, with a minimum incidence of 1 in 14,700 live births [1]. The gene mutated in the disease (*ABCD1*) encodes the ALD protein (ALDP), a peroxisomal transporter that imports very long-chain fatty acids (VLCFA) into the peroxisome for degradation [2, 3]. Elevated plasma VLCFA is a pathognomonic biomarker for this disorder, although it lacks predictive value for disease severity or progression [4]. Different phenotypes have been described in X-ALD. Virtually all patients who reach adulthood develop the most frequent phenotype, adrenomyeloneuropathy (AMN), in their third and fourth decades of life. The initial symptoms are limited to the spinal cord and peripheral nerves. Patients develop progressive spastic paraparesis, sensory ataxia with impaired vibration sense, sphincter dysfunction, pain in the legs, and impotence [5]. Neurophysiological findings reveal axonal neuropathy and disturbances in evoked potentials [6]. Brain magnetic resonance imaging (MRI) results are often abnormal, mainly affecting the corticospinal tract [7]. Approximately 80% of patients suffer from adrenocortical insufficiency [5]. Women carriers often present mild myelopathy after the fourth decade of life [8]. The most severe disease phenotype, cerebral childhood adrenoleukodystrophy (cALD), presents rapidly progressive inflammatory brain demyelination with a lethal outcome unless diagnosed early and treated with hematopoietic bone marrow transplant [9] or the new available hematopoietic stem cell therapy [10].

At present, there is no satisfactory treatment for patients with AMN [11]. Oxidative stress is a major factor driving X-ALD pathogenesis [12–17] and appears very early in life [12]. At the origin of this, redox imbalance is a combination of increased production of mitochondrial radical oxygen species (ROS) caused by the excess of VLCFA [18], together with a deficient endogenous antioxidant response [13, 19]. The combination of antioxidants used herein, α-tocopherol (vit E), N-acetylcysteine (NAC), and α-lipoic acid (LA), has been shown to be synergistic *in vitro*, halting the clinical progression and axonal damage in a murine model of AMN [20]. Moreover, this combination ameliorates key metabolic pathways contributing to the pathogenetic cascade, such as energy production [21], mitochondrial biogenesis and respiration [22], proteostasis [23, 24], and endoplasmic reticulum stress [25]. These findings indicate that oxidative damage is a very early driver of pathogenesis, whereas providing a strong rationale for clinical translation. To date, no biomarkers of disease progression have been identified in X-ALD, as the VLCFA levels in plasma do not correlate with the onset or severity of symptoms. The present work contributes to filling this knowledge gap.

## Methods

### Study Design and Subjects

This was a phase II pilot, prospective, open-label, single-center study, which was approved by the Spanish Agency of Medicines and Medical Devices and by the Clinical Research Ethics Committee of Bellvitge University Hospital and was registered at ClinicalTrials.gov (NCT01495260). The trial was conducted in accordance with the ethical standards of the Declaration of Helsinki. The subjects were followed up for 2 years (8 visits). Thirteen subjects, 12 men and 1 woman, were selected for the study. The age of the subjects ranged from 24 to 64 years. All the subjects had a confirmed diagnosis of AMN on the basis of elevated VLCFA levels and an identified pathogenic variant of the *ABCD1* gene. All the subjects met all the inclusion criteria and none of the exclusion criteria in accordance with the protocol.

#### Inclusion Criteria

Symptomatic subjects over 18 years of age, with confirmed diagnosis of AMN by elevation in VLCFA and the presence of a mutation in the *ABCD1* gene.

#### Exclusion Criteria

Cerebral inflammatory disease verified with gadolinium enhancement, cerebral disease with cognitive impairment, Expanded Disability Status Scale (EDSS) > 6.5, hypersensitivity to compounds related to cysteine, peptic ulcer, asthma, severe respiratory failure, impaired hepatic function, or other basic blood and urine tests, and pregnant, lactating, or childbearing-aged women.

Clinical and demographic data for the subjects at the initiation of the study are available in Table 1. VLCFA levels and pathogenic variants in *ABCD1* of the subjects are available in Supplemental Data (Table 1).

**Table 1:** Clinical and demographic data

PAT	Age (years)	Sex	Dose	EDSS	MS UL	MS LL	SPAS UL	SPAS LL	DTR UL	DTR LL	CLON UL	CLON LL	CPR	SENS
1	24	M	B	6.0	5/5	4/5	1	3	2/4	3/4	–	AC	EXT	AP
2	47	M	B	6.0	5/5	5/5	1	2	3/4	3/4	–	–	EXT	N
3	35	M	B	1.0	5/5	5/5	1	2	2/4	2/4	–	–	FLX	AP
4	44	M	B	4.5	5/5	5/5	1	3	3/4	3/4	–	AC	EXT	AP
5	45	M	B	5.0	5/5	4/5	2	3	2/4	3/4	–	AC	EXT	AP
6	37	M	B	6.5	5/5	5/5	2	3	2/4	3/4	–	+	EXT	HP
7	51	M	B	6.0	5/5	4/5 distal	1	3	3/4	3/4	–	AC	EXT	HP
8	41	M	A	6.0	5/5	4/5	1	3	3/4	3/4	–	AC	EXT	HP
9	28	M	B	4.5	5/5	4/5	1	2	3/4	3/4	–	–	EXT	HP
10	38	M	B	4.0	5/5	4/5 distal	1	2	3/4	3/4	–	AC	EXT	AP
11	37	M	B	2.0	5/5	5/5	1	3	3/4	3/4	–	+	EXT	HP
12	64	F	B	4.5	5/5	5/5	1	3	2/4	3/4	–	–	EXT	HP
13	31	M	B	4.0	5/5	4+/5 distal	1	3	3/4	3/4	–	AC	EXT	HP

PAT = patient; M = male; F = female; EDSS = Expanded Disability Status Scale; MS = muscular strength; UL = upper limbs; LL = lower limbs; SPAS = spasticity; DTR = deep tendon reflexes; CLON = clonus; AC = achilles clonus; CPR = cutaneous-plantar reflex; FLX = flexor; EXT = extensor; SENS = sensibility; AP = apallesthesia; HP = hypopallesthesia; N = normal vibratory sensibility

For statistical purposes, plasma and peripheral blood mononuclear cells (PBMCs) from 25 healthy age- and sex-matched individuals and cerebrospinal fluid (CSF) from 9 subjects with nonrelated diseases were used as controls.

### Procedures

**Fig. 1:**
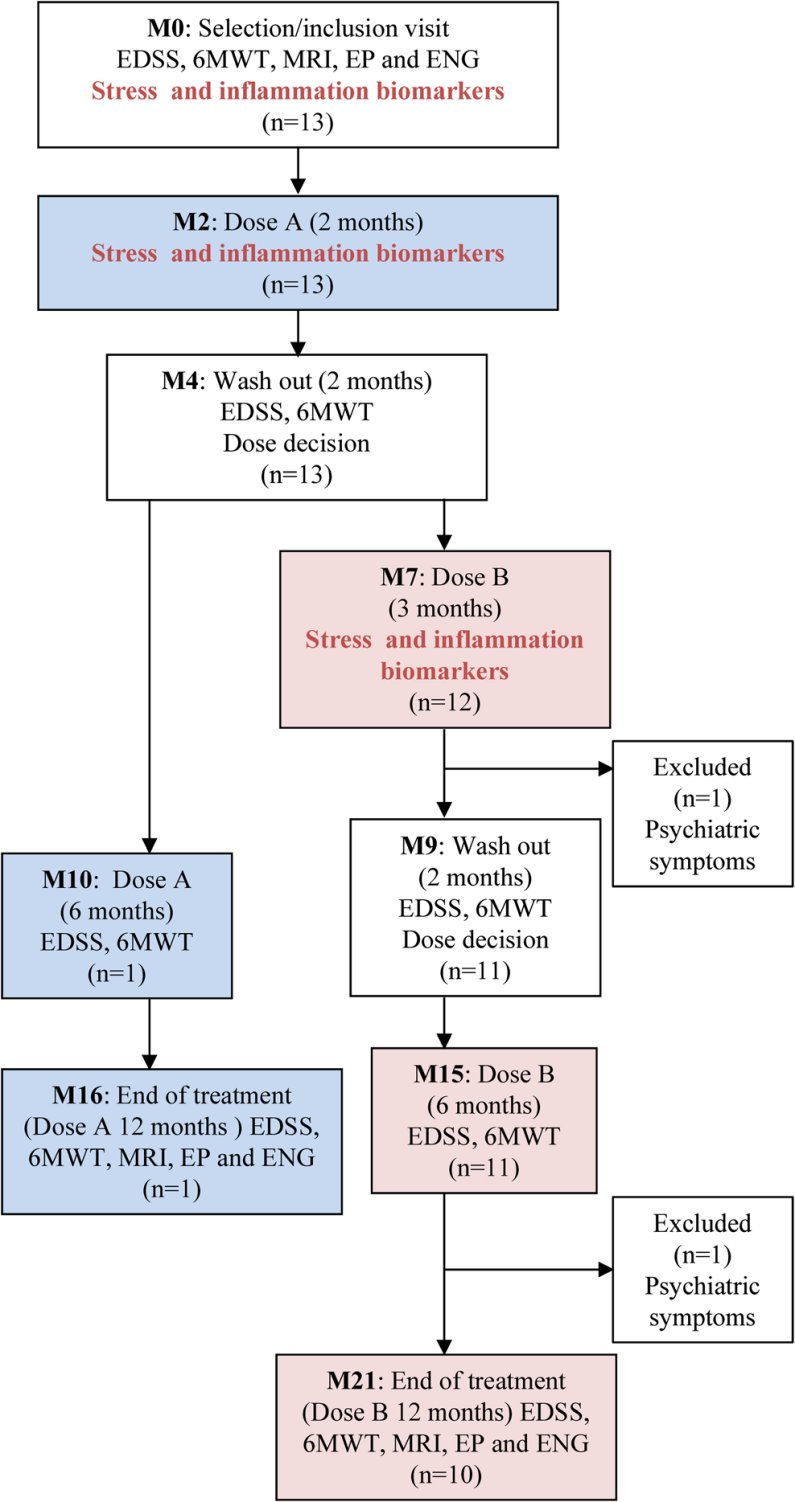
Trial profile. Patients initially received a lower oral dose A daily for 2 months (M2). After that, a 2-month washout period was introduced during which the biomarkers of protein oxidative damage were tested in plasma (M4). In patients showing normalization of biomarkers (patient 8), the treatment was restarted for 12 months at the same dose (M10, M16). In patients showing no normalization of oxidative damage biomarkers, the dosage was increased to dose B for 3 months (M7). After this treatment period, the biomarkers were tested again during a new washout period of 2 months (M9). If normalization of the levels was attained with dose B, the treatment was restarted for 12 more months with the higher dose (M15, M21). In the eventuality that protein oxidative damage biomarkers were not reduced, the patient would have been considered a nonresponder, and the treatment would have been discontinued at that point. This eventually did not apply to any of the patients. Blue boxes, patients taking dose A; pink boxes, patients taking dose B

Because this combination of antioxidants has not, to our knowledge, been used previously, we used 2 different doses owing to safety considerations. Patients initially received a lower dose A: NAC (800 mg), LA (300 mg), and vit E (150 IU) orally daily for 2 months. After this treatment period, a 2-month washout period was introduced, and protein oxidative damage biomarkers in plasma were tested. In patients showing normalization of biomarkers, the treatment was restarted for 12 months at the same dose. In patients showing no normalization of oxidative damage biomarkers, the dosage was increased to dose B for 3 months: NAC (2400 mg), LA (600 mg), and vit E (300 IU), single high doses already reported in the literature. After this treatment period, the biomarkers were tested again. A further washout period of 2 months ensued. If normalization of the levels was attained with dose B, the treatment was restarted for 12 more months with the higher dose. In the eventuality that protein oxidative damage biomarkers were not reduced, the patient was considered a nonresponder, and the treatment was discontinued at that point. Plasma and PBMC extractions were assessed at all visits (Fig. 1).

Only 1 patient (patient 8) exhibited normalized oxidative damage biomarkers in plasma and PBMCs with the lower dose of antioxidants (dose A). Thus, and following the protocol, the patient was maintained on the same treatment regimen (dose A) for an additional year until the end of the study. The remaining subjects were treated with dose B for an additional year.

### Primary Outcomes

#### Oxidative Protein Damage Markers

Metal-catalyzed oxidation products: AASA (aminoadipic semialdehyde) and GSA (glutamic semialdehyde); mixed glycoxidation/lipoxidation products: CEL (Nepsilon-(carboxyethyl)-lysine) and CML (Nepsilon-(carboxymethyl)-lysine); and lipid peroxidation-derived products: MDAL (Nepsilon-malondialdehyde-lysine) were quantified in the plasma as previously reported [12]. AASA, CML, CEL, and MDAL were expressed as micromoles per mole lysine.

#### Oxidative DNA Damage Marker

8-Oxo-dG (7,8-dihydro-8-oxo-2-deoxyguanosine) was tested in the urine by HPLC according to the methodology described by Haghdoost et al. [26]. The 8-oxo-dG content was expressed as nanogram per milligram creatine.

The reference values for oxidative damage biomarkers were calculated according to the sample distribution of 25 healthy individuals. The point that defines a superior moderate outlier in the sample of controls was taken as the reference limit (Q75 + 1.5 × IQR, Q75 = 75% percentile, IQR = interquartile range).

#### Inflammatory Biomarkers

We quantified the following molecules with previously reported methods [27]: i) plasma eicosanoids and oxidized polyunsaturated fatty acids (AA, DHA, PGD2, PGE2, PGF2α, 6-keto-PGF1α, 9S-HODE, 13S-HODE, 12S-HETE, 15S-HETE, and TXB2); ii) plasma inflammatory cytokines, chemokines, and signalling receptors (HGF, IL-6, IL-8, MCP1, TNF, and adiponectin); iii) PBMC cytokines and signalling receptors (*IFNA2, IL-37, IL-4, PPARα, IL-10, IL-36A, IL-36RN, IL-13, CCR3, CXCL5, IL-9R,* and *STAT1*). Briefly, eicosanoids and oxidized polyunsaturated fatty acids were measured by a triple quadrupole mass spectrometry–based metabolite quantification assay (Biocrates Life Science AG, Innsbruck, Austria); HGF, IL-6, IL-8, MCP1, TNF, and adiponectin were determined by immunoassays using the Milliplex kit (Luminex xMAP Technology, EMD Millipore Corporation, Billerica, MA), and *IFNA2, IL-37, IL-4, PPARα, IL-10, IL-36A, IL-36RN, IL-13, CCR3, CXCL5, IL-9R,* and *STAT1* gene expression was quantified by real-time PCR with Taqman® probes in total RNA from PBMCs. We also tested neopterin in the cerebrospinal fluid according to the methodology described by Molero-Luis et al. [28].

### Secondary Outcomes

#### Clinical Outcomes

All the subjects were clinically examined using a standardized neurologic examination, including central motor, sensory, cerebellar, cerebral, and peripheral nerve motor functions. A standard neurologic history and examination were used to score the subjects on the Kurtzke EDSS [29]. The 6-minute walk test (6MWT) [30] was performed at all follow-up visits. For statistical purposes, this test was considered at 0 versus 12 months.

#### Evoked Potentials

Visual evoked potentials (VEP), brainstem auditory evoked potentials (BAEP), motor evoked potentials (MEP), somatosensory evoked potentials (SEP), and laser-evoked potentials (LEP) were obtained through standard techniques using Synergy electromyographs (Oxford Instruments, Surrey, UK). Normative data were obtained from 10 healthy adults examined under the same experimental conditions [31]. Abnormality was defined as the presence of a latency, amplitude, or conduction velocity > 2.5 SD above the mean values in healthy adults.

#### VEP

Monocular pattern reversal VEP was recorded by means of a pattern reversal black-and-white checkerboard presented at 1.9 Hz on a cathode ray tube screen. The visual angle was 35′ and 70′. Monocular stimulation was performed by covering 1 eye with an eye patch, and full-field stimuli were given. The recording electrode was placed at Oz according to the international 10/20 system and referenced to Fz. The ground electrode was placed over the Cz. Signals were recorded using Synergy electromyographs (Oxford Instruments) with a bandpass filter set at 1 to 100 Hz, with a sweep time of 500 ms. At least 2 averages of 100 artifact-free trials were recorded. We measured P100 latency and amplitude.

#### BAEP

BAEPs were obtained for clicks presented at 70 dB above the hearing threshold using a Synergy electromyograph (Oxford Instruments). A click sound was presented to the unilateral ear on the reference side at a rate of 21.1 Hz using headphones; each side was examined separately. The bandpass filter was set at 100 Hz to 1.5 kHz. Evoked responses of 1500 stimuli were averaged, and at least 2 trials for each ear were conducted. The recording electrode was placed over the vertex (Cz), the reference electrode was placed on the unilateral earlobe, and the ground electrode was placed over the Fz. The peak latencies of I, II, III, IV, and V waves were measured, and the interpeak latency between I, III, and V waves was calculated.

#### MEP

During the magnetic stimulation, the patients lay comfortably in a supine position. MEPs were recorded from the abductor digiti minimi of the hypothenar and abductor hallucis muscles of the foot using Ag/AgCl surface cup electrodes placed in a belly tendon montage. Signals were recorded using Synergy electromyographs (Oxford Instruments) with filters set at 3 Hz and 10 kHz. Magnetic stimulation was conducted using a monophasic stimulator (Magstim 200; The Magstim Co. Ltd., UK) and a round magnetic stimulating coil (10-cm diameter; The Magstim Co. Ltd.) to stimulate upper limbs and a double cone coil (11-cm diameter; The Magstim Co. Ltd.) to stimulate lower limbs. The stimulus was applied over the vertex of the scalp. To obtain preferential activation of each hemisphere, induced current flowed from the posterior to the anterior direction over the motor area. We measured the threshold for evoking a response in the target muscles. Responses were recorded at rest and with a minimum voluntary contraction (facilitation), because MEP size increases and its latency shortens when the muscle is voluntarily contracted. We recorded 4 consecutive responses at rest and with facilitation and measured the response with a minimum latency and maximum amplitude. The central motor conduction time (CMCT) was calculated with the following formula: CMCT = CM−((DL + *F* wave−1) / 2), in which CM is the latency between the cortex and the muscle explored, DL is the distal latency of the motor response, and *F* is the shortest *F* wave latency.

#### SEP

For SEP recording, patients lay in the supine position in a warm and semidarkened room to make the patient as comfortable as possible and help him to relax. SEPs were elicited after electrical stimulation (0.2-ms duration, constant current, 4.13-Hz stimulus rate) with skin electrodes from both median nerves at the wrist and posterior tibial nerves at the ankle. The intensity of electrical stimuli was set slightly above the motor threshold. The number of sweeps averaged was between 500 and 1000. Samples with excessive interference were automatically rejected from the average. The EP tracings were replicated and superimposed to demonstrate the reproducibility of the components measured. The filter bandpass was 3 to 3000 Hz. The analysis time was 50 ms for median nerve SEPs and 100 ms for tibial nerve SEPs. Recording electrodes for the median nerve SEPs, with an electrical impedance of less than 4 kΩ, were placed bilaterally in the supraclavicular fossa (Erb’s point), in the skin overlying the sixth cervical spinous process (Cv6) and in the parietal scalp region (C3′ and C4′). The Erb’s point electrode was referred to the contralateral Erb’s point, the Cv6 electrode was referred to an electrode located immediately above the thyroid cartilage and scalp electrodes (C3′ and C4′) were referred to Fz. For the posterior tibial nerve SEPs, the recording electrodes were placed over the tibial nerve in the popliteal fossa, over the low back on the skin overlying the spinous processes of vertebra L1, over the skin overlying the cervical spine and in Cz′ (2 cm posterior to the Cz) in the scalp. The popliteal fossa electrode was referred to an electrode placed 3 cm proximally, the first lumbar vertebra electrode was referred to an electrode located immediately above the umbilicus, the Fz electrode was referred to cervical electrode, and Cz′ was referred to Fz. For median nerve SEPs, we evaluated peaking latencies of Erb’s point N9, generated by the brachial plexus volley and recorded by the Erb’s electrode; the spinal N13 response, generated within the cervical gray matter; and contralateral scalp N20, recorded from the parietal electrode and generated in the primary somatosensory (SI) area. We also calculated N9-N13, N9-N20, and N13-N20 interpeak latencies and measured the peak-to-peak amplitude between N20 and the subsequent positive peak (P25). For tibial nerve SEPs, we evaluated the peaking latencies of the N8 recorded at the popliteal fossa, N22 recorded at L1 generated by the lumbosacral dorsal horn neurons, P30 recorded at the sixth cervical spinous process, and the scalp P40 response, probably generated in the SI area. We also calculated N22-P40 to assess the conduction time in central somatosensory pathways and measured the peak-to-peak amplitude between P40 and the subsequent negative peak (N50).

#### LEP

LEPs were obtained by delivering brief radiant heat pulses to the face, dorsum of the hand and leg by means of a NdYAP laser stimulator (wave length 1.34 μm; beam diameter 5 mm; energy 1.5–3.5 J; duration 5 ms). The stimulus intensity was approximately 1.5 to 2 times the mean pain threshold of healthy subjects. Signals were recorded using a Synergy electromyograph (Oxford Instruments). LEPs were recorded from Cz with reference to linked earlobes, using silver/silver chloride cup electrodes of 9-mm diameter filled with a conductive adhesive gel. The amplifier bandpass frequency filter was 0.2 to 100 Hz. The analysis time was 1 s. Impedance was maintained at less than 4 kΩ. Ten artifact-free LEPs were recorded for each side and averaged offline. We measured the latency of the first negative peak (N2) and of the subsequent positive peak (P2), as well as the peak-to-peak amplitude (N2/P2 amplitude).

#### Nerve Conduction Studies

Synergy electromyographs (Oxford Instruments) were used for these studies. The following parameters were measured: nerve conduction velocity, distal latency (with the distance kept constant), and amplitude of response. Recordings were performed using standard methods [32]. Abnormality was defined as the presence of a latency, amplitude, or conduction velocity > 2 SD or 1 SD. In motor nerve conduction studies, at least 1 unilateral median, ulnar, and peroneal nerve was evaluated. Distal and proximal compound muscle action potential (CMAP) amplitude, distal motor latency (DML), duration of proximal and distal CMAP (dCMAP), and motor nerve conduction velocity (MNCV) were also assessed. The sensory tests were performed in the sural nerve, except in 1 patient (patient 7), who had suffered a previous bilateral sural biopsy. In this patient, the studies were performed in the superficial peroneal nerve. The motor nerve conduction studies were performed in the peroneal nerve except for patient 1, for whom the studies were performed in the tibial nerve. With regard to these studies, 7/13 subjects presented a mild peripheral neuropathy at the beginning of the trial. A pattern suggestive of neuropathy with demyelinating features was observed in 2/13 subjects, and axonal neuropathy in 5/13 subjects.

#### MRI Outcomes

MRI with gadolinium contrast was performed in a 1.5-T apparatus (Philips Healthcare, Best, The Netherlands). Diffusion tensor imaging (fractional anisotropy maps) was obtained for each patient. Sagittal T1-weighted, coronal-enhanced IRT1-weighted imaging, and FLAIR sequences were obtained for the axial plane and coronal sequences with pulse inversion recovery providing high contrast between white matter lesions and other adjacent structures. The subjects were classified into 3 groups according to severity of the lesions in FLAIR/T1 images. Three subjects (3, 8, and 9) showed a normal pattern, 7 exhibited moderate lesions in the corticospinal tract (CST) and peritrigonal white matter (mild hyperintensity on FLAIR sequence, with a bilateral and symmetrical distribution), and 3 (subjects 1, 4, and 6) exhibited clearly pathological lesions in the CST. In those 3 subjects, the lesions were more severe in the internal capsule and midbrain, peritrigonal white matter, optic radiation, and corpus callosum; in addition, the pattern of lesions was more focal, with prominent altered signals.

DTI was acquired using a single-shot sequence echo planar imaging. The image matrix was 144 and a field of vision (FOV) of 234 × 170 mm was the direction of phase AP. The entire skull was studied, including stem, with 60 axial sections 2 mm thick, which were acquired parallel to the anterior and posterior white matter commeasured with isometric voxel 2 × 2 × 2 mm. The value of *b* was 400 to 800 s/mm^2^ with an average resolution, and 16 points were calculated. From this sequence, the isotropic image and fractional anisotropy (FA) maps for each of the patients were obtained. Regions of interest (ROIs) were defined coinciding with MRS voxel, and the FA values were obtained at the bilateral corticospinal tract. A third ROI was performed in 3 patients in whom impaired signal was observed in the white matter on conventional sequences. Visual inspection of the diffusion images was performed to exclude moving images or artifacts. The single-voxel ^1^H-MRS technique was used to acquire data from MRS. A VOI from 1.5 to 2 cm^3^ was placed in the left parieto–occipital white matter. Two spectra were acquired in the same VOI: 1) SE STE (TR/TE/stockings, 2000/30/96 -192) and 2) (TR/TE/averages, 2000/136/128-256) SE LTE. Myo/Cr, Cho/NAA, and NAA/Cr ratios were calculated for each patient. The same radiologist performed the evaluation of the images and calculations consecutively.

#### Safety Outcomes

Two patients were removed from the statistical analysis. Subject 1, who showed a more severe lesion pattern on MRI among the patients, presented a behavioral change after 9 months in the trial, with a jocular attitude, puerility, and impulsiveness, signs of frontal release (glabellar and palmomental reflexes), and developed inflammatory lesions 6 months after the end of the treatment. Subject 6 presented hypomania, perseveration, and disinhibition, as previously noted in the pre-existing medical history, although the principal investigator considered during the inclusion period that he would be able to follow through with treatment. Although the development of psychiatric disease and cerebral manifestations is common in AMN, because we did not include an untreated comparison group, we cannot formally exclude an unlikely treatment-related event. Furthermore, the low number of patients and short observation period in our cohort preclude from extrapolating how many would be expected to develop the cerebral AMN phenotype in our cohort.

### Statistical Analysis

Protein oxidative damage, inflammation biomarkers, and Q-PCR array expression data were examined for normality by the Shapiro–Wilk test. Significant differences were determined using a 1-tailed Student’s *T* test if the data were normally distributed or a 1-tailed Wilcoxon rank sum test otherwise. When analyzing the treatment effect by comparing patient scores at different time points, we used the 1-tailed paired Student’s *T* test if the data were normally distributed or the 1-tailed Wilcoxon signed rank test otherwise. We chose the 1-tailed *T* test instead of the 2-tailed because we aimed to elucidate whether or not the analyzed parameter deviated from the controls in the same sense for all subjects. We further analyzed whether these values were affected by the treatment and changed in concert as a block in a given sense. The patient on dose A was excluded from the statistical study, as the dose used was much lower than the rest of patients.

Principal component analysis (PCA) was applied as previously reported [33]. We performed 3 different analyses grouping the variables under the following categories: 1) clinical variables and 2) expression levels of inflammatory markers and mediators. We considered those factors that changed significantly in response to treatment, and we analyzed their levels prior to treatment as variables, together with the age of the patients. To extract the eigenvalues and calculate the factor loadings, PCA was performed with the S-Plus princomp function in the R programming environment.

Penalty regression methods were conducted using the glmnet package [34] in R. Briefly, we applied a likelihood ratio test using the lmtest package [35] to differentiate the influence of the variables selected by the penalty regression methods from confounding variables such as age and distance walked in the 6MWT before treatment [36]. The statistical analyses were performed using the Bioconductor packages in the R programming environment [37]. To investigate whether the distance walked at the end of the assay in the 6MWT could be predicted by the levels of oxidative damage and inflammatory markers, we distributed the data into 3 groups of variables: i) inflammatory cytokines, chemokines, and receptors; ii) inflammation-associated lipids; and iii) the expression of inflammatory cytokines, chemokines, and receptors. We also included the age and 6MWT distance for each patient before treatment as independent variables. We applied *Y* = *b*_0_ + *b*_1_*X*_1_ + *b*_2_*X*_2_ … *b*_n_*X*_n_ to the independent variables (*X*_1_… *X*_n_), which were defined as the 6MWT distance before treatment, age, MCP1, and 15S-HETE levels, to better predict the dependent variable (*Y*), i.e., the 6MWT distance at the end of the study. To validate the penalized regression model, the analysis included a leave-one-out cross-validation (LOOCV) procedure characterized by using a single sample as validation data for testing the model, whereas the remaining samples were utilized as training data. The cross-validation process was then repeated as many times as the number of samples used in the model [38].

## Results

### Primary Outcomes

A main purpose of this study was to identify and validate robust biomarkers to monitor the biological effects and efficacy of the proposed combination of antioxidants, and thereby lay groundwork for future, larger trials with drugs targeting redox or inflammatory homeostasis.

#### Oxidative Stress Markers

Our primary objective aimed to pinpoint a dose of antioxidants that was safe and biologically active to normalize the quantitative markers of oxidative damage to proteins previously tested in a small number of patients with AMN [39], hence validating these biomarkers. We also included a well-characterized marker of oxidative damage to DNA, 8-oxo-dG, which is currently broadly used for neurodegenerative disorders [40, 41].

**Fig. 2:**
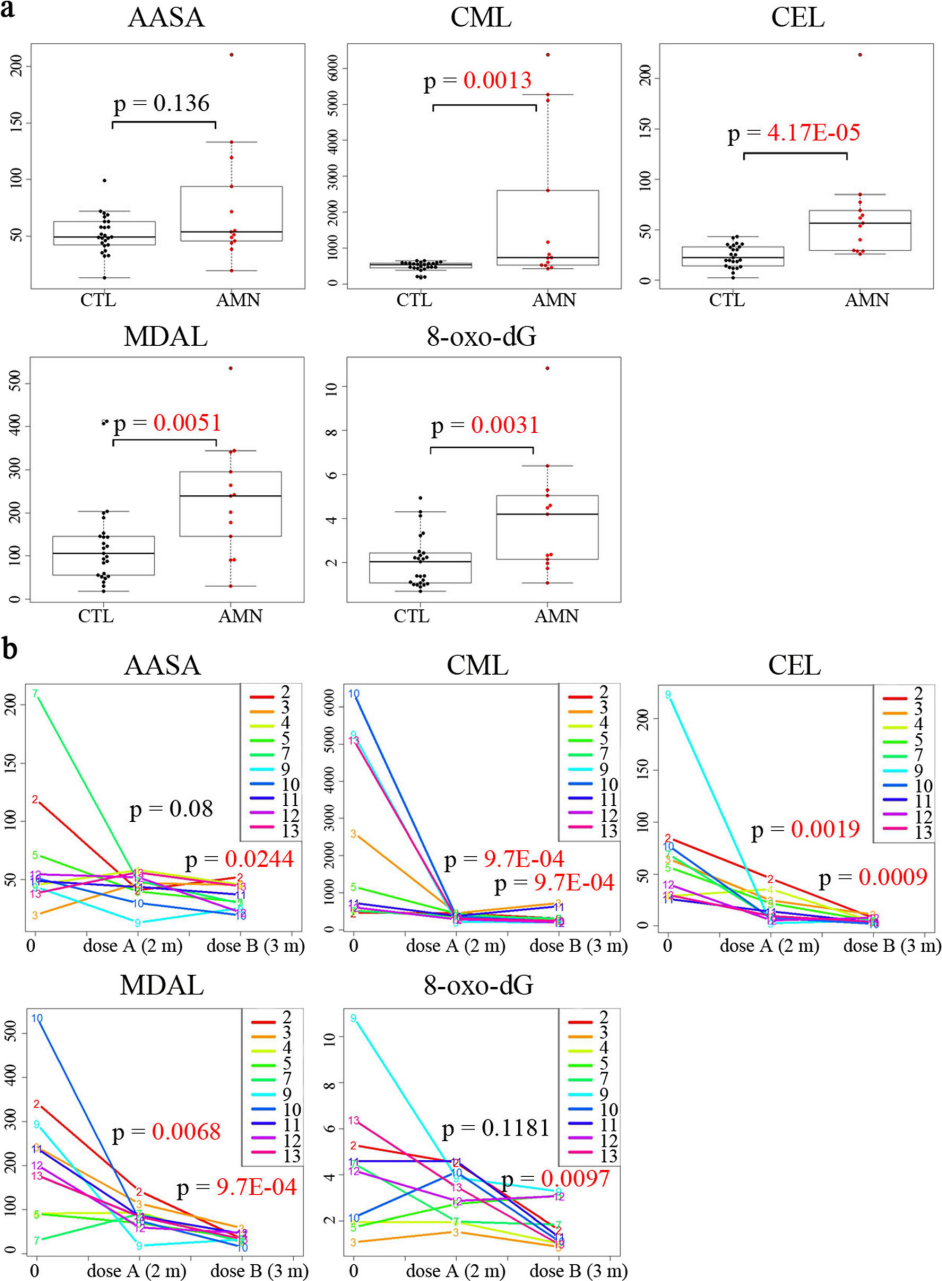
Oxidative lesion markers and treatment effect in subjects. **(a**) Significant pretreatment increase in the oxidation markers CML, CEL, MDAL in the plasma, and 8-oxo-dG in the urine is observed in subjects compared with controls. Whiskers indicate 1.5 times the interquartile range; the bottom and top of the boxes indicate the first and third quartiles, respectively; the center lines of the boxes indicate the second quartile. (**b**) A significant decrease was observed in the plasma concentrations of the oxidation markers CML, CEL, and MDAL after 2 months with dose A (M2) and, more strikingly, after 3 months with dose B (M7) compared with pretreatment levels at the initial time point (M0). A significant decrease in AASA in plasma and urine 8-oxo-dG levels was only observed after 3 months with dose B. *P* values are colored red if they are less than 0.05. AASA, CML, CEL, and MDAL are expressed as μmol/mol lysine; 8-oxo-dG is expressed as ng/mg creatine

At baseline, we observed a significant increase in CML, CEL, MDAL, and 8-oxo-dG levels (in plasma and urine, respectively) and a trend towards higher AASA levels in the plasma of AMN patients compared with sex- and age-matched controls, with CEL levels being the best discriminating factor of genotype (Fig. 2a). After treatment with dose A (2 months), the levels of AASA and 8-oxo-dG did not vary, with the exception of patient 8, who showed normalized levels. The remaining subjects received dose B after a washout period of 2 months, and this higher dose was found to drastically decrease all oxidative damage markers after 3 months of use (Fig. 2b). Patient 12, the only female patient included in the study, presented similar values to those detected in male patients.

#### Inflammatory Markers

**Fig. 3:**
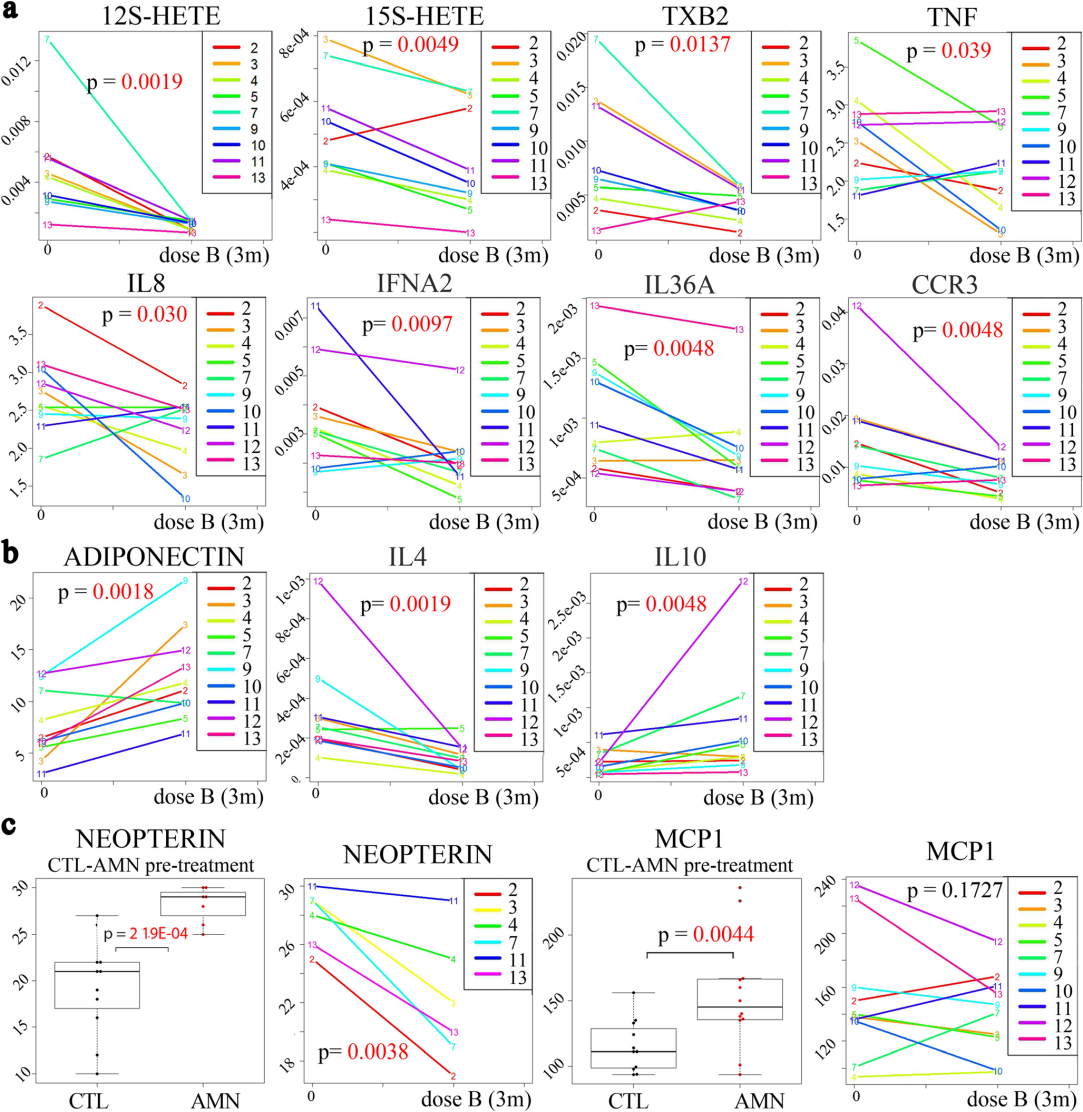
Effects of the antioxidant treatment on inflammatory markers**.** (**a**) A significant decrease was observed in the pro-inflammatory markers 12S-HETE, 15S-HETE, TXB2, TNF, IL-8, IFNA2, IL-36A, and CCR3 after 3 months with dose B compared with pretreatment levels. (**b**) A significant increase was detected in the levels of the anti-inflammatory markers adiponectin and IL-10 after 3 months with dose B compared with pretreatment levels at the initial time point (time 0). The same treatment led to a significant decrease in *IL-4* levels. (**c**) Significantly higher levels of neopterin in cerebrospinal fluid and MCP1 in plasma were observed in AMN patients before treatment, and neopterin levels exhibited a significant decrease after 3 months of treatment. Whiskers indicate 1.5 times the interquartile range; the bottom and top of the boxes indicate first and third quartiles, respectively; the center lines of the boxes indicate the second quartile. *P* values are colored red if they are less than 0.05. The 12S-HETE, 15S-HETE, and TXB2 in plasma and neopterin in cerebrospinal fluid are expressed as μmol/l; TNF, IL-8, and MCP1 in plasma are expressed as pg/ml; adiponectin in plasma is expressed as μg/ml; *IFNA2, IL-36A, CCR3, IL-4,* and *IL-10* are expressed as the relative gene expression in PBMCs

We had previously used an integrated omics approach in plasma and PBMCs of patients with AMN, identifying several molecules of potential interest [27]. Patients with AMN exhibited significantly higher plasma levels of the lipid mediators of inflammation, 12S-HETE, 15S-HETE, and TXB2, as well as the cytokines and inflammation-related proteins, IL-6, TNF, IL-8, HGF, and MCP1, and lower levels of the protective protein adiponectin. At the mRNA level, we also reported significant overexpression of the inflammatory markers *IFNA2, IL-37, IL-4, IL-10, IL-9R, IL-36RN, IL-36A, IL-13, CCR3, CXCL5, *and *STAT1* [27]. Here, we could validate these molecules in an independent cohort, and we found that antioxidant treatment significantly decreased the levels of pro-inflammatory *IFNA2, IL-4, IL-36A*, and *CCR3* in PBMCs, and 12S-HETE, 15S-HETE, TXB2, TNF, and IL-8 in plasma, and increased those of anti-inflammatory plasma adiponectin relative to pretreatment levels. In a similar manner, the treatment further increased the already-high levels of the anti-inflammatory cytokine *IL-10* in PBMCs. In addition, we also tested neopterin, a sensitive marker of immune system activation for neurodegenerative disorders such as Parkinson’s disease [42], which was not previously reported in X-ALD. We found elevated levels of neopterin in the CSF before treatment; these levels were significantly decreased with antioxidant treatment in all the subjects from whom CSF was available (Fig. 3).

#### Safety Outcomes

Both high and low doses of the combination of antioxidants were safe and well-tolerated. Four subjects experienced mild adverse events during the trial in relation to mild and transient constipation and responded well to conservative dietary treatment. Subjects 1 and 6 interrupted the treatment after 9 and 3 months, respectively, because the principal investigator had doubts regarding fulfillment of the treatment given their behavioral compromise.

### Secondary Outcomes

#### Clinical Outcomes

**Fig. 4:**
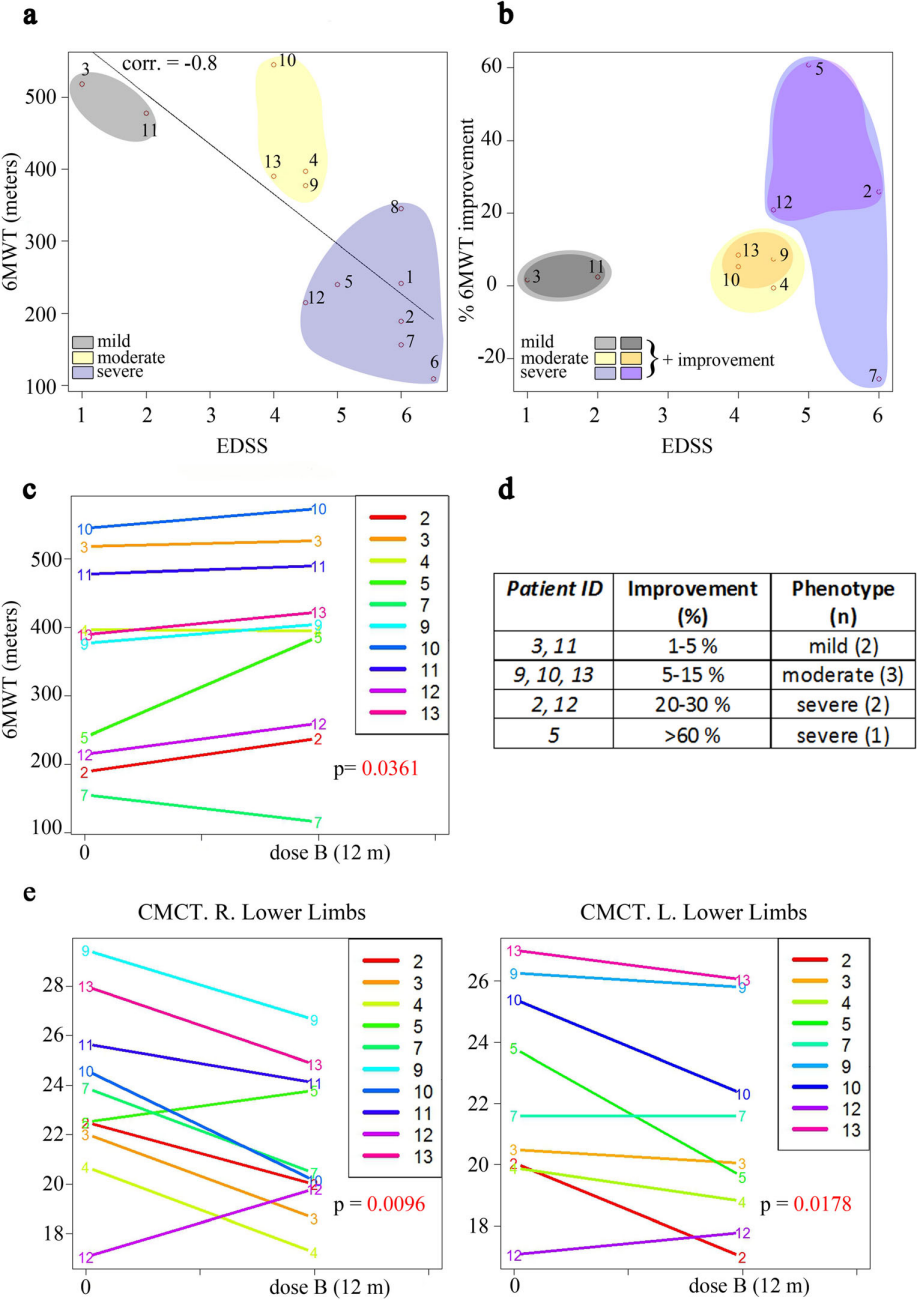
Effects of treatment on the 6-minute walk test (6MWT) and on motor evoked potentials. (**a**) The correlation between the 6MWT and EDSS before treatment at the initial time point. (**b**) The correlation between the 6MWT and EDSS at the end of treatment. In (**a**) and (**b**), the colors represent the 3 different phenotypes: mild in gray, moderate in yellow, and severe in blue. Subjects with an improvement are represented by darker colors. The correlation was measured using Pearson’s product moment correlation coefficient test. (**c**) Significant improvements were observed in the distance walked in the last visit compared with the pretreatment distances. Eight subjects improved in the distance walked, 1 showed no change and 1 worsened. (**d**) The percentage of improvement of subjects in the 6MWT is indicated. Only subjects with amelioration of their scores are listed. *n* = number of cases of each phenotype. (**e**) A significant decrease was observed in central motor conduction time (CMCT) in both legs in the last visit in comparison with baseline. R = right; L = left. *P* values are colored in red if they are less than 0.05. CMCT is the difference between the shortest peripheral motor and the shortest corticomotor latencies

Because disturbances in walking and gait are key components of the AMN clinical presentation, ambulation-related outcome measures were chosen as the most relevant clinical endpoints. The 6MWT is considered an accurate, reproducible, simple-to-carry out, well-tolerated, and well-established outcome measured in a variety of diseases [30]. Importantly, the 6MWT assesses function and endurance, which are important aspects of AMN disease status. Before treatment, we observed a strong inverse correlation between the EDSS and 6MWT distance (Pearson’s correlation, 0.8; associated probability = 0.001). According to the patient distribution in this correlation representation (Fig. 4a), we therefore grouped the subjects into 3 clinical phenotypes: mild (EDSS between 1 and 2 and high 6MWT distances) (gray), moderate (EDSS between 3 and 4.5 and intermediate 6MWT distances) (yellow), and severe (EDSS between 4.5 and 6 and low 6MWT distances) (blue) (Fig. 4a). At the end of the study, the EDSS scores remained stable in all subjects. However, we found an overall significant improvement of the distance walked in the 6MWT that varies depending on the clinical status of the patients at M0 (Fig. 4b). The distance walked increased in 8 subjects, did not change for 1 patient, and worsened by 20% in patient 7 (Fig. 4c). Subjects with a more severe presentation improved between 20 and 60% (subjects 2, 5, and 12), those in the moderate group improved slightly (5–15%, subjects 9, 10, and 13), and those with a milder presentation improved less than 5% (subjects 3, 11) (Fig. 4b, d).

#### Electrophysiological Outcomes

All the subjects presented with alterations in MEP and BAEP, 10/11 presented with disturbances in SEP, and 1/13 in VEP at baseline [31]. We also measured LEP, which provides a direct functional examination of the nociceptive afferents, particularly neuropathic pain [43]. We found that LEP signals increased in 8/13 subjects [31]. After 12 months of treatment with antioxidants, although the values were still above normal, we detected a significant decrease in the central motor conduction time of MEP in both legs, thus reflecting a slight improvement in upper motor neuron function (paired *t* test, right *p* = 0.0096, left *p* = 0.0178) (Fig. 4e). The rest of the evoked potentials remained statistically unchanged, although 1 patient exhibited normalized LEPs after treatment (details in Tables 2a, b in Supplemental Data). Regarding nerve conduction studies, none of the subjects exhibited differences in these values with respect to baseline after 1 year of antioxidants (details in Table 3 in Supplemental Data).

#### MRI Outcomes

DTI analysis revealed that all the subjects exhibited lower fractional anisotropy (FA) values in the bilateral corticospinal tract, in particular patients 6, 7, and 10 at baseline, although the differences were not significant statistically. At the end of the antioxidant treatment, no significant differences were observed (details in Table 4 in Supplemental Data).

### Stratification of Subjects by Integrating Biomarkers and Clinical Variables

**Fig. 5:**
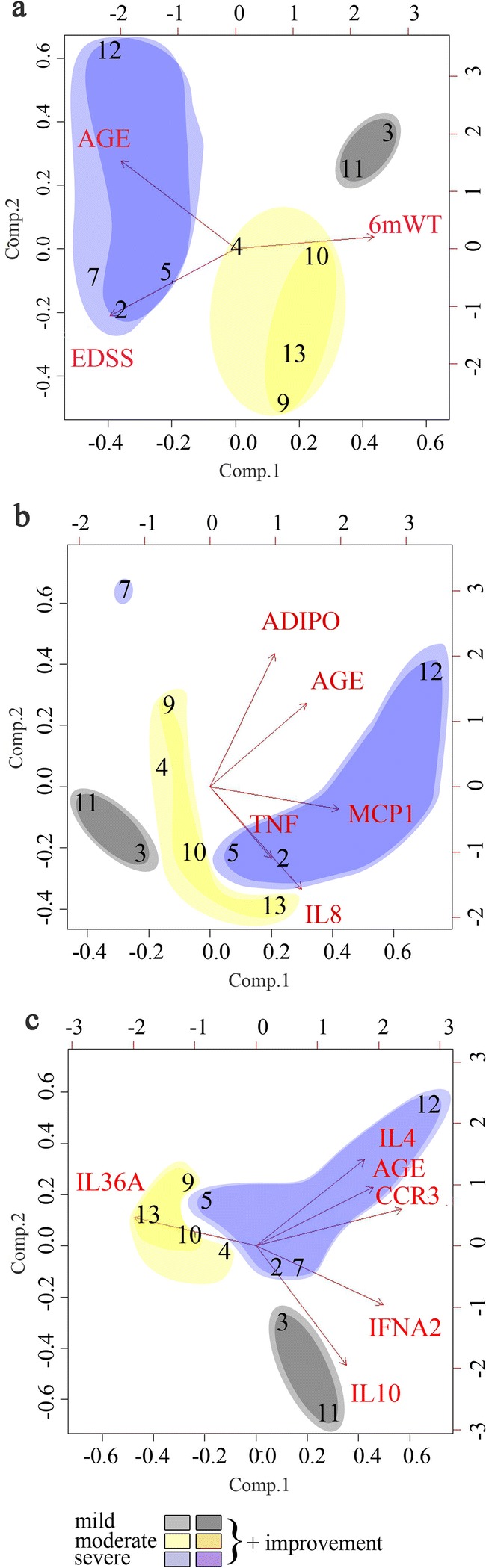
Stratification of patients by integrating biomarkers with clinical variables. PCA was used to distribute patients with AMN according to their clinical phenotype severity (in (**a)**) and according to their pretreatment biomarker values (**b**, **c**). Loading plots: the axes for components 1 and 2 indicate the most varying direction of the data, and the arrows show the direction of the variables. Mild phenotypes are shown in gray, moderate phenotypes in yellow, and severe phenotypes in blue. Darker colors depict patients who improved clinically in the 6MWT after treatment. In (**a**), the clinical variables EDSS and 6MWT are shown in relation to age. In (**b**), the plasma levels of inflammatory molecules in relation to age are shown. The most severe phenotypes show higher levels of MCP1, TNF, and IL-8, except for patient 7. In (**c**), the expression of molecules and mediators of inflammation in PBMCs are shown. Patients 3 and 11, with milder phenotypes, showed higher levels of protective IL-10

We applied PCA to examine whether subjects with AMN could be stratified into subgroups according to their clinical severity. Figure 5 shows the relationship among specific biomarkers, which are represented as a vector, and the patient’s distribution with respect to these vectors according to the biomarker values of each patient prior to treatment (baseline, M0). We observe that patient distribution matches the phenotype classification as defined previously in Fig. 4a, in groups according to severe (blue), moderate (yellow), and mild (gray) EDSS scores/6MWT at M0. In this sense, we can see in Fig. 5a how the distance measured at 6MWT correlates inversely with age and EDSS. We also see how the most severe patients with higher EDSS (2, 5, and 7) are distributed towards the end of the EDSS vector and how mild patients (3 and 11) are located near the end of the 6MWT vector. In Fig. 5b, the most severe patients are distributed towards the end of their vectors representing the pro-inflammatory markers MCP1, TNF, or IL-8, whereas the milder patients (3 and 11) are just at the opposite end of their vectors. This means that, taken together, the consideration of the levels of MCP1, TNF, and IL-8 helps to stratify the patients according to clinical severity. Patient 7, who showed a severe phenotype, is an exception, but it is worth noting that he did not improve in the 6MWT (Fig. 5b). We also observed that subjects in the mild subgroup presented with higher *IL-10* levels (patients 3 and 11) (Fig. 5c). Remarkably, the treatment did not normalize the anti-inflammatory cytokine *IL-10* but instead increased its expression, thus suggesting that the regulation of *IL-10* involves redox-sensitive mechanisms and may be protective in the X-ALD context.

### Association of MCP1 and 15S-HETE with the 6MWT

**Fig. 6:**
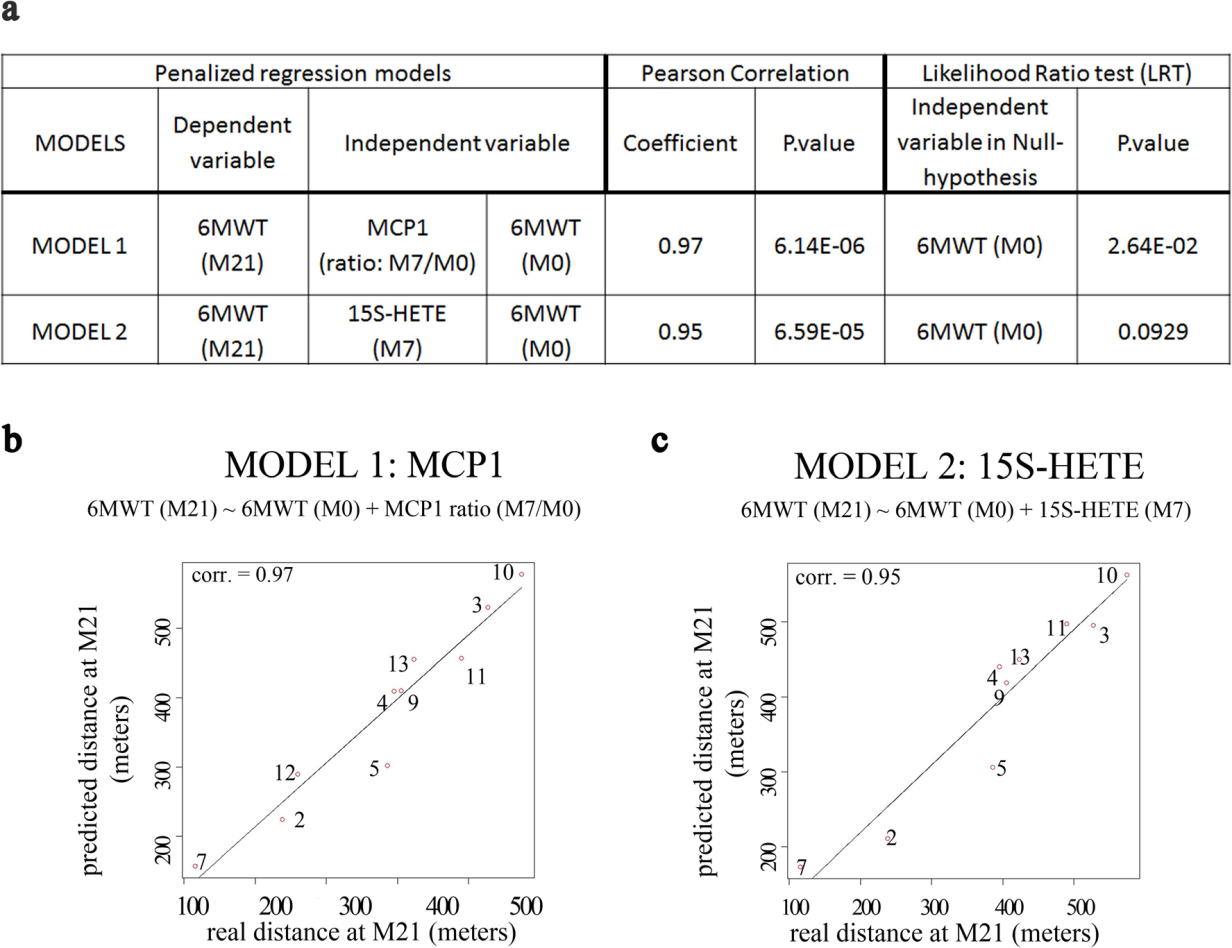
Association of 15S-HETE and MCP1 with the 6MWT. (**a**) Penalized regression models showing the best clinical improvement predictors based on the final distance walked by the subjects after treatment. (**b**, **c**) Predicted distances versus the real distances at the end of the study for the MCP1 and 15S-HETE regression models, respectively, based on Pearson’s correlation. The numbers in circles indicate the patient code

We applied penalized regression methods to investigate whether the distance walked at the end of the study (M21) in the 6MWT could be predicted by the levels of oxidative damage and inflammatory markers, either at baseline or at M7. Figure 6 shows the models with a maximum of 2 predictors that best fitted the clinical outcomes. We present 2 different models in Fig. 6a (model 1 and model 2), which consider the 6MWT before treatment at M0, and the MCP1 ratio levels at the M7/M0, or the 15S-HETE levels at the M7 visit, respectively, as independent variables. A significant inverse association was observed between the MCP1 ratios (M7 visit/M0), and the distance walked in the 6MWT for each patient, compared with his own baseline. These results indicated that subjects who showed a more prominent decrease in MCP1 levels at M7 also showed a more marked improvement in walking distance at the end of the treatment. The goodness of fit of the model is confirmed by the very significant Pearson correlation between the predicted and the real meters walked at the M21 (Pearson’s correlation, 0.97; *P* = 6.14E−06), Fig. 6a, b. Another model that was successful in predicting 6MWT improvement was the one that included the 15S-HETE levels at the M7 visit (Pearson’s correlation, 0.95; *P* = 6.59E−05) (Fig. 6a, c). Specifically, 15S-HETE levels at M7 were inversely proportional to the improvement in distance walked in the 6MWT at the end of the study. This result indicated that the lower the levels of the MCP1 ratio or 15S-HETE levels at M7, the more important the amelioration in walking capacity at the end of the study. In other words, 15S-HETE and, in particular, MCP1 ratios are predictive of response to treatment, both positive and negative. It is worth noting that the levels of none of the single biomarkers at M0 are significantly associated with the clinical status, neither at M0 nor with the improvement in the 6WMT at M21. We have found that the MCP1 or the 15S-HETE levels standing alone are insufficient to predict the clinical efficacy of the treatment. The clinical status of the patient at baseline (6MWT at time M0) needs to enter the equation as well, as deduced from the statistical analysis using penalized regression models. On the other hand, the *p* value of the likelihood ratio test (LRT, Fig. 6a) indicates that the 6MWT at M0 standing alone is less powerful predicting the 6MWT at M21 than the combination of 6MWT at M0 plus the MCP1 ratios. The same is true for model 2 including 15S-HETE, although, in this case, it does not reach statistical significance, indicating that model 1 is the most robust predictive model in this set of patients.

## Discussion

This open-label trial was primarily envisaged to translate the positive preclinical results obtained on a mouse model of adrenomyeloneuropathy treated with the same combination of antioxidants, by identifying a safe, well-tolerated dose that would achieve biological efficacy in patients. The design also aimed to identify and validate meaningful biomarkers to monitor biological efficacy or target engagement to guide larger, randomized placebo-controlled studies. We believe this objective was successfully achieved and provide herein a panel of quantitative oxidative lesion markers and lipid inflammatory mediators in the plasma, cytokines in the plasma and PBMCs, neopterin in the CSF, and 8-oxo-dG in the urine, which may be useful for monitoring future treatments and disease progression. Most importantly, we report the normalization of several inflammation markers upon antioxidant treatment, in addition to markers of oxidative damage, indicating that redox and inflammatory homeostasis are deeply intertwined in this disease.

Indeed, the pro-inflammatory derivatives of arachidonic acid (TBX2, 12S-HETE, and 15S-HETE) and several pro-inflammatory cytokines and chemokines (IL-8, TNF, *IFNA2, IL-4, IL-36A*, and *CCR3*) exhibited greatly decreased or normalized values after the antioxidant treatment. Of note, the treatment increased the levels of the protective cytokine adiponectin, which was previously reported to be significantly diminished in a small cohort of subjects with AMN [27] and a mouse model [44]. Furthermore, antioxidant treatment increased the already-elevated levels of the anti-inflammatory cytokine *IL-10*, suggesting a protective effect of the antioxidants in this series. Although AMN has long been considered to be a non-inflammatory disorder—as opposed to cerebral childhood ALD or adult-onset cerebral AMN—mounting evidence indicates that pro-inflammatory cascades are significant although controlled under tight regulatory cues [27]. On this pro-inflammatory background, which may represent the “default form” of the disease, additional genetic or environmental hits, such as brain trauma, may provoke conversion to the rapidly progressive and lethal cerebral ALD or AMN forms [45].

In this trial, we have learned that redox homeostasis is directly linked to inflammatory status in this disease, so that antioxidants may help to ameliorate the chronic, low-grade pro-inflammatory state that characterizes AMN and, as a consequence, modify or slow disease progression. It is tempting to speculate that our results may strengthen the rationale for the improved outcomes of hematopoietic stem cell transplantation in the presence of NAC [46] and argue for the use of antioxidants as a companion treatment also for the most severe cerebral inflammatory forms of the disease, cALD. In addition, using penalized regression methods, we discovered markers with predictive value for the response to treatment. Indeed, the levels of MCP1 negatively affected the percentage of improvement in the walked distance in the 6MWT, indicating that several subjects showed a correlation between an improvement in walking distance and a decrease in MCP1 levels following treatment. The reverse was also true for patient 7, who showed increased MCP1 levels and a poorer 6MWT result in the final visit. Thus, the response of MCP1 to the antioxidant treatment (by decreasing its levels in comparison with baseline) may be considered a candidate predictor of disease progression in this cohort. Of note, MCP1 has been shown to be increased in the CSF of children with cALD [47], underscoring the interest in this biomarker across discordant disease phenotypes. This chemokine is the most potent inducer of the signal transduction pathways leading to monocyte transmigration [48, 49], and accumulating evidence suggests that MCP1 and its receptor, CCR2, may be prime targets to combat neuroinflammation [50]. The same conclusion applies to 15S-HETE, a lipid mediator of inflammation and potent agonist of PPARbeta/delta receptors [51, 52], which showed an inverse correlation with an improvement in walking distance in this series. Thus, confirmation of the predictive value of MCP1 and 15S-HETE in additional, larger cohorts is warranted.

With all the natural, obliged reservation that emanates from a small open trial, it is intriguing that the treatment appeared to confer a statistically significant clinical benefit in several parameters. We detected a significant improvement in 6MWT, particularly in subjects with higher EDSS scores. The 6MWT appeared to be sufficiently sensitive to discern any possible differences in the distances walked and to be a good clinical outcome measure for the follow-up of moderately or severely affected subjects during the treatment.

Although a placebo effect cannot be excluded, the robust improvements in several biological outcome measures, as well as some mild but statistically significant amelioration in independent clinical and neurophysiological outcomes, may suggest an intriguing positive signal. Furthermore, this work provides a series of markers to monitor the biological effects of prospective trials, some of which, such as MCP1, have the potential to correlate with disease progression and clinical efficacy. Likewise, the 6MWT may be meaningful to monitor the clinical treatment efficacy. The observed tolerability, safety, and signs of clinical benefit of this pilot study may warrant the long-term use of these or other next-generation antioxidants [53] in a phase III, double-blind, randomized, placebo-controlled clinical trial with a larger number of subjects. The safety and tolerability of the doses used may facilitate extension of similar antioxidant combinations to other diseases that share both axonal degeneration as a significant component of clinical progression and redox imbalance as a primary or early contributing pathogenic factor [54, 55].

## Electronic Supplementary Material

ESM 1 (DOC 476 kb)

ESM 2 (PDF 647 kb)
