# Supplementary material for: Biomarker Identification, Safety, and Efficacy of High-Dose Antioxidants for Adrenomyeloneuropathy: a Phase II Pilot Study
Source: Neurotherapeutics. 2019 May 10;16(4):1167–82. doi: 10.1007/s13311-019-00735-2 (PMC6985062; doi:10.1007/s13311-019-00735-2)
Supplement: Supplementary file 1 — (DOC 476 kb) [file 13311_2019_735_MOESM1_ESM.doc]

**Supplemental Data**

Biomarker identification, safety and efficacy of a high-dose antioxidant combination for adrenomyeloneuropathy: a phase II pilot study

Carlos Casasnovas, MD^1,2,4*^ Montserrat Ruiz, PhD,^2,3,4,*^ Agatha Schlüter, PhD, ^2,3,4^ Alba Naudí, PhD,^5^ Stéphane Fourcade, PhD,^2,3,4^ Misericordia Veciana, MD,^6^ Sara Castañer, MD,^7^ Antonia Albertí, MD,^1^ Nuria Bargalló, MD,^8,9^  Maria Johnson, MS,^10^ Gerald V. Raymond, MD,^10^ Ali Fatemi, MD,^11^ Ann B. Moser, PhD,^11^ Francesc Villarroya, PhD,^12,13^ Manuel Portero-Otín, MD,^5^ Rafael Artuch, MD^4,14^ Reinald Pamplona, MD, and Aurora Pujol, MD.^2,3,15*^

|  | | | | | | | | | | | | | | | |
| --- | --- | --- | --- | --- | --- | --- | --- | --- | --- | --- | --- | --- | --- | --- | --- |
|  |  |  |  |  |  |  |  |  |  |  |  |  |  |  |  |
|  |  |  |  |  |  |  |  |  |  |  |  |  |  |  |  |
|  |  |  |  |  |  |  |  |  |  |  |  |  |  |  |  |
|  |  |  |  |  |  |  |  |  |  |  |  |  |  |  |  |
|  |  |  |  |  |  |  |  |  |  |  |  |  |  |  |  |
|  |  |  |  |  |  |  |  |  |  |  |  |  |  |  |  |
|  |  |  |  |  |  |  |  |  |  |  |  |  |  |  |  |
|  |  |  |  |  |  |  |  |  |  |  |  |  |  |  |  |
|  |  |  |  |  |  |  |  |  |  |  |  |  |  |  |  |
|  |  |  |  |  |  |  |  |  |  |  |  |  |  |  |  |
|  |  |  |  |  |  |  |  |  |  |  |  |  |  |  |  |
|  |  |  |  |  |  |  |  |  |  |  |  |  |  |  |  |
|  |  |  |  |  |  |  |  |  |  |  |  |  |  |  |  |
|  |  |  |  |  |  |  |  |  |  |  |  |  |  |  |  |

| **Table 3**. **Nerve conduction data.** pre=pre-treatment; post=post-treatment; R= Right; L= Left; SENS NCS= Sensory Nerve Conduction Studies; MOT NCS= Motor Nerve Conduction Studies; Lat= Latency; AMP= Amplitude; NCV= Nerve Conduction Velocity; NR= No Response; in bold =abnormal values | | | | | | | | | | | | | | | | | | | | | | | | | | | | | | | | | | | | | | | | | | | | |  |
| --- | --- | --- | --- | --- | --- | --- | --- | --- | --- | --- | --- | --- | --- | --- | --- | --- | --- | --- | --- | --- | --- | --- | --- | --- | --- | --- | --- | --- | --- | --- | --- | --- | --- | --- | --- | --- | --- | --- | --- | --- | --- | --- | --- | --- | --- |
|  | | | | | | | | | | | | | | | |  | | | | | | | | | | | | | | | | | | | | | | | | | | | | | |
| **ID** | **AGE** | **SIDE** | **SENS NCS (Sural Nerve)** | | | | | | | | | | | | | | | | | **MOT NCS (Peroneal Nerve)** | | | | | | | | | | | | | | | | | | | | | | | | |  |
|  |  |  | LAT (ms) | | | | | AMP(µV) | | | | | NCV (m/s) | | | | | | | LAT (MS) | | | | | | | AMP (µV) | | | | | | | NCV (m/s) | | | | | | | | | | |  |
|  |  |  |  |
|  |  |  | pre | | post | | pre | | | post | | | | pre | | | post | | | pre | | | post | | | pre | | | | post | | | pre | | | | | | | | post | | | |  |
| 1 | 24 | R | 2.4 | | | 2.65 | | 23.7 | | | | 23.5 | 58.3 | | | | | 52.8 | | 3.8 | | | | 3.65 | | | 29.9 | | | | 25.1 | | | 43.1 | | | | | | | | 49.2 | | |  |
|  |  | L | 2.45 | | | 2.75 | | 25.3 | | | | 19.6 | 57.1 | | | | | 50.9 | | 3.9 | | | | 3.3 | | | 26.1 | | | | 24.1 | | | 44.6 | | | | | | | | 43.5 | | |  |
| 2 | 48 | R | 2.65 | | | 2.9 | | 22 | | | | 21.7 | 52.8 | | | | | 49.3 | | **5.65** | | | |  | | | 8.2 | | | |  | | | 47.1 | | | | | | | |  | | |  |
|  |  | L | 2.7 | | | 2.55 | | 20.3 | | | | 21 | 51.9 | | | | | 48.3 | | 5.15 | | | | 5.4 | | | 8.9 | | | | 4.3 | | | 54.5 | | | | | | | | 49.6 | | |  |
| 3 | 35 | R | 2.6 | | | 2.65 | | 11.4 | | | | **6.6** | 53.8 | | | | | 48.3 | | 5.75 | | | | 3.65 | | | 6.2 | | | | 7.4 | | | 42 | | | | | | | | 38 | | |  |
|  |  | L | 3.1 | | | 2.95 | | **9** | | | | **6** | 45.2 | | | | | 44.5 | | 4.85 | | | |  | | | 7.3 | | | |  | | | 41.8 | | | | | | | |  | | |  |
| 4 | 44 | R | 2.5 | | | 2.35 | | **8** | | | | **4.4** | 56 | | | | | 62.2 | | 3.8 | | | | 4.2 | | | 6.4 | | | | 3.8 | | | 44 | | | | | | | | 42 | | |  |
|  |  | L | 2.4 | | | 2.35 | | **9.8** | | | | **11.3** | 58.3 | | | | | 59.6 | | 4.9 | | | |  | | | 2.6 | | | |  | | | 43.7 | | | | | | | |  | | |  |
| 5 | 45 | R | 2.8 | | | 2.3 | | **9.9** | | | | 17.7 | 51.8 | | | | | 51.2 | | 3.55 | | | | 4.3 | | | 4.2 | | | | 1.1 | | | 42.4 | | | | | | | | 40.7 | | |  |
|  |  | L | 2.7 | | | 2.35 | | **10** | | | | 19.9 | 53.8 | | | | | 50.1 | |  | | | | 4.15 | | |  | | | | 1.9 | | |  | | | | | | | | 42.2 | | |  |
| 6 | 37 | R | 2.85 | | | 2.65 | | 14.6 | | | | 14.3 | 49.1 | | | | | 47.1 | | 4.55 | | | | 4.75 | | | 2.2 | | | | 8.2 | | | 45.9 | | | | | | | | 43.7 | | |  |
|  |  | L | 2.5 | | |  | | 22.9 | | | |  | 56 | | | | |  | |  | | | |  | | |  | | | |  | | |  | | | | | | | |  | | |  |
| 7 | 50 | R | 2.85 | | | 2.55 | | 15.3 | | | | 15.3 | **41.5** | | | | | **42** | | 4.55 | | | | 4.85 | | | 8.5 | | | | 6 | | | **33.3** | | | | | | | | **32.9** | | |  |
|  |  | L | 2.5 | | | 2.4 | | 11.7 | | | | 15.9 | 48 | | | | | 46.2 | |  | | | | 4.85 | | |  | | | | 6 | | |  | | | | | | | | **33.1** | | |  |
| 8 | 41 | R | 2.75 | | | 3.35 | | **9.3** | | | | **8.2** | 54.5 | | | | | 41.8 | | 3.95 | | | | 4.75 | | | 5.3 | | | | 7.1 | | | 41.9 | | | | | | | | 38.8 | | |  |
|  |  | L | 2.9 | | | 3.3 | | **10.3** | | | | **8.1** | 50 | | | | | 42.4 | |  | | | | 4.9 | | |  | | | | 4.1 | | |  | | | | | | | | 37.5 | | |  |
| 9 | 28 | R | 2.3 | | | 2.2 | | 15.7 | | | | 15 | 60.9 | | | | | 63.6 | | 4.95 | | | | 3.35 | | | 5.5 | | | | 7.9 | | | 43.1 | | | | | | | | 45.6 | | |  |
|  |  | L | 2.6 | | |  | | 9.9 | | | |  | 53.7 | | | | |  | |  | | | |  | | |  | | | |  | | |  | | | | | | | |  | | |  |
| 10 | 38 | R | 2.75 | | |  | | 17.6 | | | |  | 50.9 | | | | |  | | 6.3 | | | | 5.3 | | | 10.6 | | | | 5 | | | 42.2 | | | | | | | | 43.5 | | |  |
|  |  | L | 2.15 | | | 2.6 | | 21.4 | | | | 14.7 | 60.5 | | | | | 53.8 | |  | | | |  | | |  | | | |  | | |  | | | | | | | |  | | |  |
| 11 | 37 | R | 2.35 | | | 2.85 | | **9.8** | | | | **11.1** | 59.6 | | | | | 49.1 | | 4.55 | | | | 4.45 | | | 7.8 | | | | 3.9 | | | 43.8 | | | | | | | | 44.6 | | |  |
|  |  | L | 2.45 | | |  | | **7.3** | | | |  | 57.1 | | | | |  | |  | | | |  | | |  | | | |  | | |  | | | | | | | |  | | |  |
| 12 | 64 | R | **NR** | | | **NR** | | **NR** | | | | **NR** | **NR** | | | | | **NR** | | 4.2 | | | | 4.8 | | | **1.2** | | | | **1.3** | | | 48.8 | | | | | | | | 48 | | |  |
|  |  | L | **NR** | | | **NR** | | **NR** | | | | **NR** | **NR** | | | | | **NR** | |  | | | |  | | |  | | | |  | | |  | | | | | | | |  | | |  |
| 13 | 31 | R | 2.75 | | | 2.95 | | 15.5 | | | | 20.8 | 50.9 | | | | | 47.5 | | **5.85** | | | | **6.25** | | | 2.4 | | | | 3.5 | | | 24.3 | | | | | | | | **22.5** | | |  |
|  |  | L | 2.55 | | | 3 | | 19.3 | | | | 24.8 | 54.9 | | | | | 46.7 | | **6.75** | | | | **6.5** | | | 2.3 | | | | 3.4 | | | 25.2 | | | | | | | | **24.6** | | |  |
| **NORMATIVE VALUES** | | | |  | | | | |  | |  | | | |  | | | |  | |  | | | | | | |  | | | |  | | | |  | | | | | | | |  | |
| **Peroneal Nerve** | | | |  | | | | |  | | **Amp** | | | |  | | | | **Lat** | |  | | | | | | | **NCV** | | | |  | | | |  | | | | | | | |  | |
| Upper limit (mean + 2SD) Range 23-35 y-o | | | | | | | | |  | | 5.4 +/- 3 | | | |  | | | | 3.7 +/- 1.8 | | | | | |  | | | | 49·5 +/- 10·8 | | | | | | | | | | | | | | |  | |
| Upper limit (mean + 2SD) Range 44-65 y-o | | | | | | | | |  | | 5.0 +/- 2.6 | | | |  | | | | 3.7 +/- 1.4 | | |  | | | | | | | 48·3 +/-9·2 | | | | | | | | | |  | | | | | | |
| **Sural nerve** | | | |  | | | | |  | |  | | | |  | | | |  | |  | | | | | | |  | | | |  | | | |  | | | | | | | |  | |
| Upper limit (mean + 1SD) Range 23-35 y-o | | | | | | | | |  | | 20.9 +/- 8.0 | | | |  | | | | 2.7 +/- 0.3 | | |  | | | | | | | 52·5 +/- 5·6 | | | | | | | |  | | | | | | | | |
| Upper limit (mean + 1SD) Range 44-65 y-o | | | | | | | | |  | | 17.2 +/- 6.7 | | | |  | | | | 2.8 +/- 0.3 | | |  | | | | | | | 51·1 +/- 1·3 | | | | | | | | | | |  | | | | | |
| **Tibial Nerve** | | | |  | | | | |  | |  | | | |  | | | |  | |  | | | | | | |  | | | |  | | | |  | | | | | | | |  | |
| Upper limit (mean + 2SD) | | | | | | | | |  | | 5.8 +/- 3.8 | | | |  | | | | 3.96 +/- 2.00 | | | | | | | | | 48.5 +/- 7.2 | | | | | | |  | | | | | | | |  | | |
| **Superfical Peroneal Nerve** | | | | | | | | |  | |  | | | |  | | | |  | |  | | | | | | |  | | | |  | | | |  | | | | | | | |  | |
| Upper limit (mean + 1SD) Age over 15 y-o | | | | | | | | |  | | 13.9 +/-4.6 | | | |  | | | | 2.24 +/- 0.49 | | | | | | | | | 47.3 +/- 3.4 | | | | | | | | | |  | | | | | | | |

| **Table 1**. **Diagnostic data** | | | | | | | | | | | | | | | |
| --- | --- | --- | --- | --- | --- | --- | --- | --- | --- | --- | --- | --- | --- | --- | --- |
|  |  | AMN Pre-treatment (% of total fatty acids) | | | | | | | AMN Post-treatment (% of total fatty acids) | | | | | | |
|  | **Pathogenic Variants** | **C22:0** | **C24:0** | **C26:0** | **C28:0** | **C30:0** | **C24:1(n-9)** | **C26:1(n-9)** | **C22:0** | **C24:0** | **C26:0** | **C28:0** | **C30:0** | **C24:1(n-9)** | **C26:1(n-9)** |
| EC-001 | p.Arg401Gln | 0.67% | 1.12% | 0.052% | 0.0063% | 0.0013% | 1.38% | 0.024% | 0.70% | 1.13% | 0.049% | 0.0065% | 0.0016% | 1.57% | 0.028% |
| EC-002 | p.Arg554His | 0.48% | 0.87% | 0.042% | 0.0048% | 0.0011% | 1.10% | 0.026% | 0.49% | 0.83% | 0.043% | 0.0045% | 0.0015% | 1.05% | 0.026% |
| EC-003 | p.Val457fs* | 0.71% | 1.20% | 0.049% | 0.0054% | 0.0018% | 0.75% | 0.014% | 0.73% | 1.16% | 0.050% | 0.0064% | 0.0017% | 0.65% | 0.013% |
| EC-004 | p.Tyr174Cys | 0.75% | 1.25% | 0.057% | 0.0080% | 0.0021% | 0.95% | 0.019% | 0.75% | 1.22% | 0.056% | 0.0074% | 0.0011% | 0.98% | 0.020% |
| EC-005 | p.Tyr181Cys | 0.78% | 1.24% | 0.043% | 0.0057% | 0.0016% | 0.86% | 0.012% | 0.63% | 0.98% | 0.042% | 0.0054% | 0.0018% | 0.55% | 0.011% |
| EC-006 | p.Val102Glu | 0.62% | 1.02% | 0.039% | 0.0039% | 0.0009% | 0.62% | 0.013% | 0.63% | 1.09% | 0.045% | 0.0047% | 0.0008% | 0.62% | 0.014% |
| EC-007 | p.Ser108Ter | 0.79% | 1.52% | 0.059% | 0.0071% | 0.0010% | 0.96% | 0.020% | 0.74% | 1.35% | 0.054% | 0.0054% | 0.0015% | 0.79% | 0.016% |
| EC-008 | p.Gly277Arg | 0.73% | 1.08% | 0.045% | 0.0111% | 0.0049% | 1.31% | 0.022% | 0.86% | 1.37% | 0.054% | 0.0095% | 0.0028% | 1.04% | 0.017% |
| EC-009 | p.Leu628Pro | 0.44% | 0.86% | 0.048% | 0.0048% | 0.0011% | 0.63% | 0.014% | 0.72% | 1.34% | 0.059% | 0.0062% | 0.0012% | 0.68% | 0.017% |
| EC-010 | p.Arg617Leu | 0.58% | 1.23% | 0.080% | 0.0119% | 0.0024% | 1.25% | 0.036% | 0.77% | 1.45% | 0.073% | 0.0101% | 0.0023% | 1.46% | 0.037% |
| EC-011 | p.Arg389Gly | 0.75% | 1.24% | 0.043% | 0.0047% | 0.0013% | 0.88% | 0.014% | 0.89% | 1.51% | 0.052% | 0.0044% | 0.0013% | 0.86% | 0.013% |
| EC-012 | p.Arg389Gly | 0.91% | 1.17% | 0.037% | 0.0044% | 0.0010% | 0.86% | 0.012% | 0.89% | 1.18% | 0.030% | 0.0037% | 0.0014% | 0.97% | 0.014% |
| EC-013 | p.Tyr181Ter | 0.72% | 1.21% | 0.057% | 0.0071% | 0.0016% | 1.07% | 0.018% | 0.78% | 1.41% | 0.065% | 0.0076% | 0.0021% | 0.95% | 0.022% |
| **CONTROLS** |  |  |  |  |  |  |  |  |  |  |  |  |  |  |  |
| media (n=13) |  | **0.873%** | **0.818%** | **0.010%** | **0.0010%** | **0.00003%** | **1.05%** | **0.006%** | **0.873%** | **0.818%** | **0.010%** | **0.0010%** | **0.00003%** | **1.05%** | **0.006%** |
| SD |  | 0.054 | 0.051 | 0.000350 | 0.000094 | 0.000031 | 0.042 | 0.00036 | 0.054 | 0.051 | 0.000350 | 0.000094 | 0.000031 | 0.042 | 0.00036 |

*Novel pathogenic variant

**Table 2a. Evoked potential data. BAEP=Brainstem Auditory Evoked Potential; SEP=Somatosensory Evoked Potential; MEP=Motor Evoked Potential; LEP=Laser Evoked Potential ;pre=pre- treatment; post=post-treatment; R=right; L=left; NR=no response; in bold: abnormal values; CMCT=central motor conduction time; ND: not done; in bold= abnormal values**

| **ID** | **AGE** | **SIDE** | **MEP (ms)** | | **MEP (ms)** | | **BAEP (ms)** | | | | | | **LEP (ms)** | | | | | | **VEP (70')** | | | |
| --- | --- | --- | --- | --- | --- | --- | --- | --- | --- | --- | --- | --- | --- | --- | --- | --- | --- | --- | --- | --- | --- | --- |
|  |  |  | CMCT | CMCT | CMCT | CMCT | I-III | | III-V | | I-V | | TRIG |  | UL |  | LL |  | P100 | | P100 | |
|  |  |  |  |  |  |  |  |  |  |  |  |  | N2/P2 |  | N2/P2 |  | N2/P2 |  | lat | | ampl | |
|  |  |  | LL | LL | UL | UL |  |  |  |  |  |  | (ms) |  | (ms) |  | (ms) |  | (ms) | | (μV) | |
|  |  |  | pre | post | pre | post | pre | post | pre | post | pre | post | pre | post | pre | post | pre | post | pre | post | pre | post |
| 1 | 24 | R | **25.8** | **34.1** | **10.9** | **10.9** | **3.2** | **2.94** | **2.84** | **2.9** | **6.04** | **5.84** | 181 | 171 | 216 | 220 | **336** | **324** | 96.5 | 98.5 | 15.2 | 14 |
|  |  | L | **29.7** | **32.8** | **9.42** | **9.2** | **2.78** | **2.68** | **3.16** | **2.94** | **5.94** | **5.62** | 171 | 176 | 222 | 218 | **301** | **350** | 92.5 | 99 | 14 | 15.3 |
| 2 | 48 | R | **22.05** | **20** | **7.6** | 5.6 | **2.56** | **2.68** | 2.02 | 2 | **4.58** | **4.68** | 176 | 162 | 220 | 222 | 260 | 278 | 104.5 | 104.5 | 18.9 | 19.6 |
|  |  | L | **20.05** | **17** | **7.9** | 6.82 | **2.64** | **2.5** | 1.92 | 2 | 4.56 | 4.5 | 178 | 176 | 213 | 216 | 250 | 258 | 101.5 | 102 | 20.1 | 24.5 |
| 3 | 35 | R | **22.02** | **18.62** | **7.3** | **8.52** | **2.9** | **3.1** | **2.46** | **2.58** | **5.36** | **5.68** | 173 | 164 | 222 | 230 | 232 | 252 | 101 | 101.5 | 5 | 7.4 |
|  |  | L | **20.5** | **20.05** | 5.77 | **7.25** | **2.94** | **3.02** | **2.5** | **2.4** | **5.44** | **5.42** | 178 | 163 | 228 | 215 | 251 | 248 | 101 | 99.5 | 8.2 | 5.8 |
| 4 | 44 | R | **20.7** | **17.25** | **8.2** | 6.5 | **2.54** | **2.68** | **3.14** | **2.94** | **5.68** | **5.62** | 156 | 155 | 202 | 214 | 254 | 251 | 108.5 | 108 | 24.3 | 24.1 |
|  |  | L | **19.9** | **18.8** | **8.3** | 6.1 | **3.08** | **3.02** | **2.92** | **2.88** | **6** | **5.9** | 155 | 153 | 210 | 208 | 259 | 247 | 108 | 104.5 | 21 | 24.6 |
| 5 | 45 | R | **22.5** | **23.8** | **8.7** | **9.8** | **3.04** | **3** | **2.58** | **2.66** | **5.62** | **5.66** | 198 | 189 | 233 | **263** | **NR** | **NR** | 98 | 102 | 13.7 | 17.2 |
|  |  | L | **23.8** | **19.6** | **12.2** | **10.5** | **2.7** | **3** | **2.5** | **2.42** | **5.2** | **5.4** | 198 | 190 | 229 | **267** | **NR** | **NR** | 99.5 | 101 | 10 | 17 |
| 6 | 37 | R | **NR** | **28.7** | **11.8** | **11.5** | **3.12** | **3.12** | **2.96** | **2.8** | **6.08** | **5.92** | **246** | 191 | **280** | **264** | **NR** | **NR** | **118** | **115** | 2.3 | 1.7 |
|  |  | L | **NR** | **23.1** | **9.7** | **8.22** | **3.1** | **3.18** | **3.04** | **2.82** | **6.14** | **6** | **245** | 205 | **277** | **259** | **NR** | **NR** | **114.5** | **118** | 3.9 | 2.1 |
| 7 | 50 | R | **23.9** | **20.45** | **8.52** | **8.25** | 2.4 | 2.4 | 2.32 | **2.38** | **4.72** | **4.78** | 197 | 197 | **265** | **268** | **NR** | **NR** | 102 | 104 | 14.8 | 10.4 |
|  |  | L | **21.6** | **21.6** | **8.25** | **7.6** | **2.78** | **2.52** | 2.2 | 2.28 | **5** | **4.8** | 185 | 186 | **273** | 252 | **NR** | **NR** | 104 | 103.5 | 13.8 | 10.4 |
| 8 | 41 | R | **27.4** | **30.12** | **10.35** | **13.35** | **3** | **3.04** | **2.82** | **2.82** | **5.82** | **5.86** | 209 | 198 | **268** | 237 | **389** | **345** | 105 | 108.5 | 8.9 | 17.4 |
|  |  | L | **27.6** | **31.52** | **9.75** | **8.65** | **3.22** | **2.92** | **2.88** | **2.98** | **6.1** | **5.9** | 199 | 196 | **262** | 245 | **333** | **388** | 102 | 107.5 | 20.7 | 16.9 |
| 9 | 28 | R | **29.4** | **26.65** | **8.9** | **8.85** | **2.8** | **2.82** | **2.54** | **2.5** | **5.34** | **5.32** | 159 | 158 | 198 | 194 | **361** | **347** | **131** | **115.9** | 2.5 | 3.9 |
|  |  | L | **26.25** | **25.8** | **9.02** | **9.3** | **2.9** | **3** | **2.62** | **2.46** | **5.52** | **5.46** | 167 | 179 | 187 | 194 | **357** | **349** | **121** | **121.5** | 6.8 | 6.3 |
| 10 | 38 | R | **24.6** | **20.15** | **9.85** | **8.35** | 2.24 | **2.92** | **2.92** | **2.64** | **5.88** | **5.56** | **232** | 201 | **275** | 225 | **318** | 277 | 107 | **110.5** | 6.3 | 5.4 |
|  |  | L | **25.4** | **22.32** | **11.6** | **9.82** | **2.86** | **2.86** | **2.6** | **2.64** | **5.46** | **5.5** | **237** | 199 | **256** | 225 | **290** | 255 | 106 | **110** | 8.1 | 9.6 |
| 11 | 37 | R | **25.65** | **24.1** | **9.55** | **9.5** | **2.76** | **2.81** | **2.7** | **2.9** | **5.46** | **5.71** | 176 | 171 | 231 | 220 | **NR** | **NR** | 106.5 | 109.5 | 12.3 | 14.4 |
|  |  | L | **NR** | **28.9** | **8.45** | **8.12** | **2.8** | **2.85** | **2.52** | **2.98** | **5.32** | **5.83** | 190 | 197 | 219 | 213 | **NR** | **NR** | 107 | 105.5 | 12.7 | 13 |
| 12 | 64 | R | **17.07** | **19.8** | **7.95** | **8.8** | **2.72** | **2.78** | **2.42** | 2.24 | **5.14** | **5.02** | 180 | 177 | 245 | 246 | **NR** | **NR** | **111** | **112** | 18.6 | 15.6 |
|  |  | L | **17.07** | **17.8** | **8.1** | **8.2** | 2.42 | **2.58** | **2.44** | 2.14 | **4.86** | **4.72** | 162 | 176 | 236 | 238 | **NR** | **NR** | **114.5** | **116.5** | 18.3 | 17.4 |
| 13 | 31 | R | **28** | **24.85** | **12.45** | **10.1** | **3.26** | **3.4** | **2.96** | **2.86** | **6.22** | **6.26** | 182 | 182 | 233 | 229 | 280 | 254 | 101.5 | 108 | 7.5 | 5.4 |
|  |  | L | **27** | **26.05** | **9.3** | **8.65** | **3.34** | **3.3** | **2.6** | **2.58** | **6** | **5.88** | 183 | 181 | 243 | 220 | **293** | 273 | 104.5 | 106 | 5.3 | 7.7 |
| Upper limit of normative values | | | 15,7 | | 7 | | 2.47 | | 2.35 | | 4.57 | | 206 | | 255 | | 283 | | 110 | | 26.4 | |
| (mean + 2.5 SD) | | |  |  |  |  |  |  |  |  |  |  |  |  |  |  |  |  |  |  |  |  |

**Table 2b**. **Evoked potential data.** SEP=Somatosensory Evoked Potential; pre=pre-treatment; post=post-treatment; R=right; L=Left; NR=no response; in bold: abnormal values; ND: not done

| **ID** | **AGE** | **SIDE** | **SEP LL (ms)** | | | | | | | | **SEP UL (ms)** | | | | | | | | | |
| --- | --- | --- | --- | --- | --- | --- | --- | --- | --- | --- | --- | --- | --- | --- | --- | --- | --- | --- | --- | --- |
|  |  |  | N8 | | N22 | | P40 | | N22-P40 | | N9 | | N13 | | N20 | | N9-N13 | | N13-N20 | |
|
|  |  |  | pre | post | pre | post | pre | post | pre | post | pre | post | pre | post | pre | post | pre | post | pre | post |
| 1 | 24 | R | 8 | 8.2 | 22.8 | 23.1 | **NR** | **NR** | ND | ND | 10.6 | 11 | **15.75** | **15.7** | **24.15** | **25** | **5.15** | **4.7** | **8.4** | **9.3** |
|  |  | L | 8.2 | 8.2 | 23.1 | 23.1 | **NR** | **NR** | ND | ND | 10.45 | 10.65 | **14.8** | **14.75** | **24.3** | **24.4** | **4.35** | 4.1 | **9.5** | **9.7** |
| 2 | 48 | R | 8.5 | 8.2 | 23.9 | 23.9 | **49.8** | **48.5** | **25.9** | **24.6** | 10.7 | 10.5 | **15.15** | **14.8** | 22.05 | 21.4 | **4.45** | 4.3 | 6.9 | 6.6 |
|  |  | L | 8.8 | 8.4 | 23.7 | 23.9 | **49.8** | **49** | **26.1** | **25.1** | 10.5 | 10.25 | **15.5** | **14.45** | 21.4 | 21.3 | **5** | 4.2 | 5.9 | 6.85 |
| 3 | 35 | R | **10.8** | **10.5** | **29.8** | **29.8** | **47** | **48.3** | 17.2 | 18.5 | **12.6** | **12.2** | **16.75** | **17.25** | 23 | **23.6** | 4.15 | **5.05** | 6.25 | 6.35 |
|  |  | L | **10.2** | **10.5** | **29** | **29.4** | **46.8** | **47.7** | 17.8 | 18.3 | **12.5** | **12.05** | **16.9** | **17.2** | 23 | 23 | **4.4** | **5.15** | 6.1 | 5.8 |
| 4 | 44 | R | 10.1 | 9.7 | **25.6** | **25.4** | **58.7** | **55.3** | **33.1** | **29.9** | 11 | 10.04 | **15.7** | **14.6** | 22.65 | 22.4 | **4.7** | **4.56** | **6.95** | **7.8** |
|  |  | L | 9.8 | 9.3 | **26.2** | **25.1** | **58.5** | **55.4** | **32.3** | **30.3** | 11 | 10.03 | **15.45** | **15.1** | 22.25 | 21.7 | **4.45** | **5.07** | 6.8 | 6.6 |
| 5 | 45 | R | **NR** | **NR** | **NR** | **NR** | **NR** | **NR** | ND | ND | 10.8 | 11 | **NR** | **NR** | **23.9** | 24.2 | ND | ND | ND | ND |
|  |  | L | **NR** | **NR** | **NR** | **NR** | **NR** | **NR** | ND | ND | 11.1 | **11.25** | **NR** | **NR** | **24.2** | 24.3 | ND | ND | ND | ND |
| 6 | 37 | R | 9.7 | 9.9 | **26** | **25.9** | **NR** | **NR** | ND | ND | **11.25** | **11.5** | **NR** | **NR** | **24.5** | **24.6** | ND | ND | ND | ND |
|  |  | L | 9.7 | **NR** | **26.1** | **24.8** | **NR** | **NR** | ND | ND | 11.15 | **11.4** | **NR** | **NR** | **24.8** | **24.6** | ND | ND | ND | ND |
| 7 | 50 | R | **NR** | **NR** | NR | **30.4** | **60.2** | **60.9** | ND | **30.5** | **12.8** | **12.85** | **NR** | **NR** | **26.45** | **26.2** | ND | ND | ND | ND |
|  |  | L | **NR** | **NR** | **30.6** | **30.2** | **60** | **60** | **29.4** | **29.8** | **12.6** | **12.25** | **NR** | **17.25** | **25.5** | **25.9** | ND | **5.0** | ND | **8.65** |
| 8 | 41 | R | **NR** | **NR** | **28.3** | **27.6** | **NR** | **58.13** | ND | **30.53** | **11.3** | **11.3** | **16.05** | **15.52** | **25.1** | **24.95** | **4.75** | 4.22 | **9.05** | **9.43** |
|  |  | L | **NR** | **NR** | **28.2** | **27.29** | **NR** | **58.33** | ND | **31.04** | 11 | 11.2 | **14.65** | **15.05** | **24.7** | **24.74** | 3.65 | 3.85 | **10.05** | **9.69** |
| 9 | 28 | R | 9.6 | 9 | **25.6** | 24.6 | **NR** | **NR** | ND | ND | 10.7 | 10.5 | 14.05 | **15.25** | **23.9** | 22.8 | 3.35 | **4.75** | **9.85** | **7.55** |
|  |  | L | 10 | 9.6 | **25.5** | 24.4 | **NR** | **NR** | ND | ND | 10.25 | 10.1 | **14.85** | 14.25 | 22.15 | 22.1 | **4.6** | 4.15 | **7.3** | **7.85** |
| 10 | 38 | R | 9.6 | 9.1 | **26.3** | **25.3** | **57.2** | **57.6** | **30.9** | **32.3** | 10.65 | 10.5 | **15.65** | **14.8** | 22.8 | 23.2 | **5** | 4.3 | **7.15** | **8.4** |
|  |  | L | 9.6 | 9.2 | **26** | **25.6** | **58.8** | **58.4** | **32.8** | **32.8** | 10.65 | 10.6 | **15.4** | **15.65** | 23 | 23.25 | **4.75** | **5.05** | **7.6** | **7.6** |
| 11 | 37 | R | 8.5 | 8.23 | 24.5 | 24.17 | **NR** | **NR** | ND | ND | 9.75 | 9.74 | **14.9** | **15.1** | 22.2 | 22.3 | **5.15** | **5.36** | **7.3** | **7.2** |
|  |  | L | 9.3 | 8.96 | **25.2** | **24.7** | **NR** | **NR** | ND | ND | 9.8 | 9.6 | **14.4** | **14.6** | 22.8 | 22.4 | **4.6** | **5** | **8.4** | **7.8** |
| 12 | 64 | R | 9.4 | 8.3 | 22.2 | 22.9 | **49.4** | **50.9** | **27.2** | **28** | 10.05 | 10.4 | **14.15** | 13.2 | 20.95 | 21.2 | 4.1 | 2.8 | 6.8 | **8** |
|  |  | L | 8 | 7.9 | 21.5 | 22.2 | **48.5** | **48.5** | **27** | **26.3** | 9.5 | 9.75 | 13.9 | 14.2 | 20.95 | 21.2 | 4.4 | **4.45** | **7.05** | **7** |
| 13 | 31 | R | **NR** | **NR** | **NR** | **NR** | **NR** | **NR** | ND | ND | **12.1** | **12.2** | **16.3** | **16.5** | **25.8** | **26.45** | 4.2 | 4.3 | **9.5** | **9.95** |
|  |  | L | **NR** | **NR** | **NR** | **NR** | **NR** | **NR** | ND | ND | **12.1** | **11.9** | **16.8** | **16.5** | **25.9** | **26.3** | **4.7** | **4.6** | **9.1** | **9.8** |
| Upper limit of normative values | | | 10.16 | | 24.63 | | 42.91 | | 20.16 | | 11.21 | | 14.3 | | 22.46 | 4.33 | | | 6.95 | |
| (mean + 2.5 SD) | | |

| **Table 5**. Spectroscopic and Diffusion Tensor Imaging data | | | | | | | | | | | |
| --- | --- | --- | --- | --- | --- | --- | --- | --- | --- | --- | --- |
| **ID** | **AGE** | **Spectroscopy** | | | | | | | | **Difusion Tensor Imaging** | |
|  |  | Myo/Cr | | NAA/Cho | | NAA/Cr | | Cho/Cr | | FA | |
|  |  | PO | | PO | | PO | | PO | | CST | |
|  |  | pre | post | pre | post | pre | post | pre | post | pre | post |
| 1 | 24 | **0.8838** | **0.9046** | 2.10 | 2.65 | **1.6879** | **1.3367** | 0.8041 | 0.5043 | 0.515269 | **0.494690** |
| 2 | 48 | **0.8387** | 0.7861 | 2.32 | 2.41 | 1.7097 | **1.3668** | 0.7359 | 0.5661 | 0.529420 | 0.528411 |
| 3 | 35 | **1.0382** | **0.8109** | **1.31** | 1.74 | **1.6279** | **1.6218** | **1.2452** | **0.9345** | 0.554577 | 0.545326 |
| 4 | 44 | 0.7874 | **0.8153** | **3.54** | 1.73 | **1.6556** | 1.8241 | 0.4677 | **1.0514** | 0.512509 | 0.479226 |
| 5 | 45 | **0.9500** | **0.8596** | 1.52 | 1.89 | 2.0600 | **1.2682** | **1.3596** | 0.6696 | 0.522925 | 0.513929 |
| 6 | 37 | **0.9652** | **1.1509** | 1.76 | 1.45 | **1.4299** | **0.9899** | 0.8133 | 0.6818 | **0.497866** | **0.4635** |
| 7 | 50 | **0.9858** | **0.9693** | 2.16 | **1.20** | **1.4871** | **1.4559** | 0.6897 | **1.2145** | **0.492247** | **0.477074** |
| 8 | 41 | 0.7809 | 0.7233 | 1.65 | 2.18 | **1.5551** | **1.3841** | **0.9416** | 0.6360 | 0.530123 | 0.526146 |
| 9 | 28 | 0.7474 | **0.8401** | **6.79** | 2.37 | **1.6173** | **1.6132** | **0.2382** | 0.6808 | 0.518413 | 0.512709 |
| 10 | 38 | **0.8376** | **1.0453** | 1.73 | **3.58** | 1.8619 | **1.4057** | **1.0754** | **0.3928** | **0.485787** | **0.491679** |
| 11 | 37 | 0.7669 | **1.2308** | 2.68 | **4.66** | **1.6889** | **1.0735** | 0.6296 | **0.2306** | 0.554720 | 0.534809 |
| 12 | 64 | **1.0243** | **0.9385** | **3.25** | **3.14** | **1.5255** | **1.3483** | 0.4699 | 0.4298 | 0.531937 | 0.506943 |
| 13 | 31 | **0.9693** | **0.9111** | **1.20** | 1.70 | **1.4559** | **1.5715** | **1.2145** | **0.9258** | not done | not done |
| mean | | 0·8904 | 0.9220 | 2.4614 | 2.3611 | 1.6433 | 1.4046 | 0.8219 | 0.6860 | 0.5205 | 0.5062 |
| SD | | 0·10 | 0.15 | 1.48 | 0.96 | 0.17 | 0.22 | 0.34 | 0.28 | 0.02 | 0.03 |
| Normative values | |  |  |  |  |  |  |  |  |  |  |
| mean | | 0·5700 | | 2.2000 | | 1.4400 | | 0.6600 | | 0.5700 | |
| (mean +/- 3 SD) | | < 0·81 | | 2.95-1.45 | | > 1.800 | | 0.42-0.90 | | > 0.51 | |
|  |  |  |  |  |  |  |  |  |  |  |  |
| FA=Fractional anisotropy; CST= Corticospinal Tract; PO=Parieto-occipital white matter; in bold: abnormal values; | | | | | | | | |  |  |  |
| in red: withdrawn from treatment; Myo: myo-inositol; Cre: creatine; Cho: choline; NAA: N-acetylaspartate | | | | | | |  |  |  |  |  |
| Normative values (spectroscopie): Garnett et al; Brain 2000;123 (Pt 7):1403-1409. | | | | | |  |  |  |  |  |  |
| Normative values (DTI): Dubey et al; Ann Neurol 2005;58:758-766. | | | | | |  |  |  |  |  |  |
