# Supplementary material for: Biomarker Identification, Safety, and Efficacy of High-Dose Antioxidants for Adrenomyeloneuropathy: a Phase II Pilot Study
Source: Neurotherapeutics. 2019 May 10;16(4):1167–82. doi: 10.1007/s13311-019-00735-2 (PMC6985062; doi:10.1007/s13311-019-00735-2)
Supplement: Supplementary file 2 — (PDF 647 kb) [file 13311_2019_735_MOESM2_ESM.pdf]

Table 1. **Diagnostic data**

		AMN Pre-treatment (% of total fatty acids)							AMN Post-treatment (% of total fatty acids)						
	**Pathogenic Variants**	**C22:0**	**C24:0**	**C26:0**	**C28:0**	**C30:0**	**C24:1(n-9)**	**C26:1(n-9)**	**C22:0**	**C24:0**	**C26:0**	**C28:0**	**C30:0**	**C24:1(n-9)**	**C26:1(n-9)**
EC-001	p.Arg401Gln	0.67%	1.12%	0.052%	0.0063%	0.0013%	1.38%	0.024%	0.70%	1.13%	0.049%	0.0065%	0.0016%	1.57%	0.028%
EC-002	p.Arg554His	0.48%	0.87%	0.042%	0.0048%	0.0011%	1.10%	0.026%	0.49%	0.83%	0.043%	0.0045%	0.0015%	1.05%	0.026%
EC-003	p.Val457fs*	0.71%	1.20%	0.049%	0.0054%	0.0018%	0.75%	0.014%	0.73%	1.16%	0.050%	0.0064%	0.0017%	0.65%	0.013%
EC-004	p.Tyr174Cys	0.75%	1.25%	0.057%	0.0080%	0.0021%	0.95%	0.019%	0.75%	1.22%	0.056%	0.0074%	0.0011%	0.98%	0.020%
EC-005	p.Tyr181Cys	0.78%	1.24%	0.043%	0.0057%	0.0016%	0.86%	0.012%	0.63%	0.98%	0.042%	0.0054%	0.0018%	0.55%	0.011%
EC-006	p.Val102Glu	0.62%	1.02%	0.039%	0.0039%	0.0009%	0.62%	0.013%	0.63%	1.09%	0.045%	0.0047%	0.0008%	0.62%	0.014%
EC-007	p.Ser108Ter	0.79%	1.52%	0.059%	0.0071%	0.0010%	0.96%	0.020%	0.74%	1.35%	0.054%	0.0054%	0.0015%	0.79%	0.016%
EC-008	p.Gly277Arg	0.73%	1.08%	0.045%	0.0111%	0.0049%	1.31%	0.022%	0.86%	1.37%	0.054%	0.0095%	0.0028%	1.04%	0.017%
EC-009	p.Leu628Pro	0.44%	0.86%	0.048%	0.0048%	0.0011%	0.63%	0.014%	0.72%	1.34%	0.059%	0.0062%	0.0012%	0.68%	0.017%
EC-010	p.Arg617Leu	0.58%	1.23%	0.080%	0.0119%	0.0024%	1.25%	0.036%	0.77%	1.45%	0.073%	0.0101%	0.0023%	1.46%	0.037%
EC-011	p.Arg389Gly	0.75%	1.24%	0.043%	0.0047%	0.0013%	0.88%	0.014%	0.89%	1.51%	0.052%	0.0044%	0.0013%	0.86%	0.013%
EC-012	p.Arg389Gly	0.91%	1.17%	0.037%	0.0044%	0.0010%	0.86%	0.012%	0.89%	1.18%	0.030%	0.0037%	0.0014%	0.97%	0.014%
EC-013	p.Tyr181Ter	0.72%	1.21%	0.057%	0.0071%	0.0016%	1.07%	0.018%	0.78%	1.41%	0.065%	0.0076%	0.0021%	0.95%	0.022%
**CONTROLS**															
media (n=13)		**0.873%**	**0.818%**	**0.010%**	**0.0010%**	**0.00003%**	**1.05%**	**0.006%**	**0.873%**	**0.818%**	**0.010%**	**0.0010%**	**0.00003%**	**1.05%**	**0.006%**
SD		0.054	0.051	0.000350	0.000094	0.000031	0.042	0.00036	0.054	0.051	0.000350	0.000094	0.000031	0.042	0.00036

*Novel pathogenic variant

Table 2a. **Evoked potential data.** BAEP=Brainstem Auditory Evoked Potential; SEP=Somatosensory Evoked Potential; MEP=Motor Evoked Potential; LEP=Laser Evoked Potential ;pre=pre- treatment; post=post-treatment; R=right; L=left; NR=no response; in bold: abnormal values; CMCT=central motor conduction time; ND: not done; in bold= abnormal values

ID	AGE	SIDE	MEP (ms)		MEP (ms)		BAEP (ms)						LEP (ms)						VEP (70')			
			CMCT LL pre	CMCT LL post	CMCT UL pre	CMCT UL post	I-III pre	I-III post	III-V pre	III-V post	I-V pre	I-V post	TRIG N2/P2 (ms) pre	TRIG N2/P2 (ms) post	UL N2/P2 (ms) pre	UL N2/P2 (ms) post	LL N2/P2 (ms) pre	LL N2/P2 (ms) post	P100 lat (ms) pre	P100 lat (ms) post	P100 ampl (μV) pre	P100 ampl (μV) post
1	24	R	**25.8**	**34.1**	**10.9**	**10.9**	**3.2**	**2.94**	**2.84**	**2.9**	**6.04**	**5.84**	181	171	216	220	**336**	**324**	96.5	98.5	15.2	14
		L	**29.7**	**32.8**	**9.42**	**9.2**	**2.78**	**2.68**	**3.16**	**2.94**	**5.94**	**5.62**	171	176	222	218	**301**	**350**	92.5	99	14	15.3
2	48	R	**22.05**	**20**	**7.6**	5.6	**2.56**	**2.68**	2.02	2	**4.58**	**4.68**	176	162	220	222	260	278	104.5	104.5	18.9	19.6
		L	**20.05**	**17**	**7.9**	6.82	**2.64**	**2.5**	1.92	2	4.56	4.5	178	176	213	216	250	258	101.5	102	20.1	24.5
3	35	R	**22.02**	**18.62**	**7.3**	**8.52**	**2.9**	**3.1**	**2.46**	**2.58**	**5.36**	**5.68**	173	164	222	230	232	252	101	101.5	5	7.4
		L	**20.5**	**20.05**	5.77	**7.25**	**2.94**	**3.02**	**2.5**	**2.4**	**5.44**	**5.42**	178	163	228	215	251	248	101	99.5	8.2	5.8
4	44	R	**20.7**	**17.25**	**8.2**	6.5	**2.54**	**2.68**	**3.14**	**2.94**	**5.68**	**5.62**	156	155	202	214	254	251	108.5	108	24.3	24.1
		L	**19.9**	**18.8**	**8.3**	6.1	**3.08**	**3.02**	**2.92**	**2.88**	**6**	**5.9**	155	153	210	208	259	247	108	104.5	21	24.6
5	45	R	**22.5**	**23.8**	**8.7**	**9.8**	**3.04**	**3**	**2.58**	**2.66**	**5.62**	**5.66**	198	189	233	**263**	**NR**	**NR**	98	102	13.7	17.2
		L	**23.8**	**19.6**	**12.2**	**10.5**	**2.7**	**3**	**2.5**	**2.42**	**5.2**	**5.4**	198	190	229	**267**	**NR**	**NR**	99.5	101	10	17
6	37	R	**NR**	**28.7**	**11.8**	**11.5**	**3.12**	**3.12**	**2.96**	**2.8**	**6.08**	**5.92**	**246**	191	**280**	**264**	**NR**	**NR**	**118**	**115**	2.3	1.7
		L	**NR**	**23.1**	**9.7**	**8.22**	**3.1**	**3.18**	**3.04**	**2.82**	**6.14**	**6**	**245**	205	**277**	**259**	**NR**	**NR**	**114.5**	**118**	3.9	2.1
7	50	R	**23.9**	**20.45**	**8.52**	**8.25**	2.4	2.4	2.32	**2.38**	**4.72**	**4.78**	197	197	**265**	**268**	**NR**	**NR**	102	104	14.8	10.4
		L	**21.6**	**21.6**	**8.25**	**7.6**	**2.78**	**2.52**	2.2	2.28	**5**	**4.8**	185	186	**273**	252	**NR**	**NR**	104	103.5	13.8	10.4
8	41	R	**27.4**	**30.12**	**10.35**	**13.35**	**3**	**3.04**	**2.82**	**2.82**	**5.82**	**5.86**	209	198	**268**	237	**389**	**345**	105	108.5	8.9	17.4
		L	**27.6**	**31.52**	**9.75**	**8.65**	**3.22**	**2.92**	**2.88**	**2.98**	**6.1**	**5.9**	199	196	**262**	245	**333**	**388**	102	107.5	20.7	16.9
9	28	R	**29.4**	**26.65**	**8.9**	**8.85**	**2.8**	**2.82**	**2.54**	**2.5**	**5.34**	**5.32**	159	158	198	194	**361**	**347**	**131**	**115.9**	2.5	3.9
		L	**26.25**	**25.8**	**9.02**	**9.3**	**2.9**	**3**	**2.62**	**2.46**	**5.52**	**5.46**	167	179	187	194	**357**	**349**	**121**	**121.5**	6.8	6.3
10	38	R	**24.6**	**20.15**	**9.85**	**8.35**	2.24	**2.92**	**2.92**	**2.64**	**5.88**	**5.56**	**232**	201	**275**	225	**318**	277	107	**110.5**	6.3	5.4
		L	**25.4**	**22.32**	**11.6**	**9.82**	**2.86**	**2.86**	**2.6**	**2.64**	**5.46**	**5.5**	**237**	199	**256**	225	**290**	255	106	**110**	8.1	9.6
11	37	R	**25.65**	**24.1**	**9.55**	**9.5**	**2.76**	**2.81**	**2.7**	**2.9**	**5.46**	**5.71**	176	171	231	220	**NR**	**NR**	106.5	109.5	12.3	14.4
		L	**NR**	**28.9**	**8.45**	**8.12**	**2.8**	**2.85**	**2.52**	**2.98**	**5.32**	**5.83**	190	197	219	213	**NR**	**NR**	107	105.5	12.7	13
12	64	R	**17.07**	**19.8**	**7.95**	**8.8**	**2.72**	**2.78**	**2.42**	2.24	**5.14**	**5.02**	180	177	245	246	**NR**	**NR**	**111**	**112**	18.6	15.6
		L	**17.07**	**17.8**	**8.1**	**8.2**	2.42	**2.58**	**2.44**	2.14	**4.86**	**4.72**	162	176	236	238	**NR**	**NR**	**114.5**	**116.5**	18.3	17.4
13	31	R	**28**	**24.85**	**12.45**	**10.1**	**3.26**	**3.4**	**2.96**	**2.86**	**6.22**	**6.26**	182	182	233	229	280	254	101.5	108	7.5	5.4
		L	**27**	**26.05**	**9.3**	**8.65**	**3.34**	**3.3**	**2.6**	**2.58**	**6**	**5.88**	183	181	243	220	**293**	273	104.5	106	5.3	7.7
Upper limit of normative values (mean + 2.5 SD)			15,7		7		2.47		2.35		4.57		206		255		283		110		26.4	

Table 2b. **Evoked potential data.** SEP=Somatosensory Evoked Potential; pre=pre-treatment; post=post-treatment; R=right; L=Left; NR=no response; in bold: abnormal values; ND: not done

ID	AGE	SIDE	SEP LL (ms)								SEP UL (ms)									
			N8		N22		P40		N22-P40		N9		N13		N20		N9-N13		N13-N20	
			pre	post	pre	post	pre	post	pre	post	pre	post	pre	post	pre	post	pre	post	pre	post
1	24	R	8	8.2	22.8	23.1	**NR**	**NR**	ND	ND	10.6	11	**15.75**	**15.7**	**24.15**	**25**	**5.15**	**4.7**	**8.4**	**9.3**
		L	8.2	8.2	23.1	23.1	**NR**	**NR**	ND	ND	10.45	10.65	**14.8**	**14.75**	**24.3**	**24.4**	**4.35**	4.1	**9.5**	**9.7**
2	48	R	8.5	8.2	23.9	23.9	**49.8**	**48.5**	**25.9**	**24.6**	10.7	10.5	**15.15**	**14.8**	22.05	21.4	**4.45**	4.3	6.9	6.6
		L	8.8	8.4	23.7	23.9	**49.8**	**49**	**26.1**	**25.1**	10.5	10.25	**15.5**	**14.45**	21.4	21.3	**5**	4.2	5.9	6.85
3	35	R	**10.8**	**10.5**	**29.8**	**29.8**	**47**	**48.3**	17.2	18.5	**12.6**	**12.2**	**16.75**	**17.25**	23	**23.6**	4.15	**5.05**	6.25	6.35
		L	**10.2**	**10.5**	**29**	**29.4**	**46.8**	**47.7**	17.8	18.3	**12.5**	**12.05**	**16.9**	**17.2**	23	23	**4.4**	**5.15**	6.1	5.8
4	44	R	10.1	9.7	**25.6**	**25.4**	**58.7**	**55.3**	**33.1**	**29.9**	11	10.04	**15.7**	**14.6**	22.65	22.4	**4.7**	**4.56**	**6.95**	**7.8**
		L	9.8	9.3	**26.2**	**25.1**	**58.5**	**55.4**	**32.3**	**30.3**	11	10.03	**15.45**	**15.1**	22.25	21.7	**4.45**	**5.07**	6.8	6.6
5	45	R	**NR**	**NR**	**NR**	**NR**	**NR**	**NR**	ND	ND	10.8	11	**NR**	**NR**	**23.9**	24.2	ND	ND	ND	ND
		L	**NR**	**NR**	**NR**	**NR**	**NR**	**NR**	ND	ND	11.1	**11.25**	**NR**	**NR**	**24.2**	24.3	ND	ND	ND	ND
6	37	R	9.7	9.9	**26**	**25.9**	**NR**	**NR**	ND	ND	**11.25**	**11.5**	**NR**	**NR**	**24.5**	**24.6**	ND	ND	ND	ND
		L	9.7	**NR**	**26.1**	**24.8**	**NR**	**NR**	ND	ND	11.15	**11.4**	**NR**	**NR**	**24.8**	**24.6**	ND	ND	ND	ND
7	50	R	**NR**	**NR**	NR	**30.4**	**60.2**	**60.9**	ND	**30.5**	**12.8**	**12.85**	**NR**	**NR**	**26.45**	**26.2**	ND	ND	ND	ND
		L	**NR**	**NR**	**30.6**	**30.2**	**60**	**60**	**29.4**	**29.8**	**12.6**	**12.25**	**NR**	**17.25**	**25.5**	**25.9**	ND	**5.0**	ND	**8.65**
8	41	R	**NR**	**NR**	**28.3**	**27.6**	**NR**	**58.13**	ND	**30.53**	**11.3**	**11.3**	**16.05**	**15.52**	**25.1**	**24.95**	**4.75**	4.22	**9.05**	**9.43**
		L	**NR**	**NR**	**28.2**	**27.29**	**NR**	**58.33**	ND	**31.04**	11	11.2	**14.65**	**15.05**	**24.7**	**24.74**	3.65	3.85	**10.05**	**9.69**
9	28	R	9.6	9	**25.6**	24.6	**NR**	**NR**	ND	ND	10.7	10.5	14.05	**15.25**	**23.9**	22.8	3.35	**4.75**	**9.85**	**7.55**
		L	10	9.6	**25.5**	24.4	**NR**	**NR**	ND	ND	10.25	10.1	**14.85**	14.25	22.15	22.1	**4.6**	4.15	**7.3**	**7.85**
10	38	R	9.6	9.1	**26.3**	**25.3**	**57.2**	**57.6**	**30.9**	**32.3**	10.65	10.5	**15.65**	**14.8**	22.8	23.2	**5**	4.3	**7.15**	**8.4**
		L	9.6	9.2	**26**	**25.6**	**58.8**	**58.4**	**32.8**	**32.8**	10.65	10.6	**15.4**	**15.65**	23	23.25	**4.75**	**5.05**	**7.6**	**7.6**
11	37	R	8.5	8.23	24.5	24.17	**NR**	**NR**	ND	ND	9.75	9.74	**14.9**	**15.1**	22.2	22.3	**5.15**	**5.36**	**7.3**	**7.2**
		L	9.3	8.96	**25.2**	**24.7**	**NR**	**NR**	ND	ND	9.8	9.6	**14.4**	**14.6**	22.8	22.4	**4.6**	**5**	**8.4**	**7.8**
12	64	R	9.4	8.3	22.2	22.9	**49.4**	**50.9**	**27.2**	**28**	10.05	10.4	**14.15**	13.2	20.95	21.2	4.1	2.8	6.8	**8**
		L	8	7.9	21.5	22.2	**48.5**	**48.5**	**27**	**26.3**	9.5	9.75	13.9	14.2	20.95	21.2	4.4	**4.45**	**7.05**	**7**
13	31	R	**NR**	**NR**	**NR**	**NR**	**NR**	**NR**	ND	ND	**12.1**	**12.2**	**16.3**	**16.5**	**25.8**	**26.45**	4.2	4.3	**9.5**	**9.95**
		L	**NR**	**NR**	**NR**	**NR**	**NR**	**NR**	ND	ND	**12.1**	**11.9**	**16.8**	**16.5**	**25.9**	**26.3**	**4.7**	**4.6**	**9.1**	**9.8**
Upper limit of normative values (mean + 2.5 SD)			10.16		24.63		42.91		20.16		11.21		14.3		22.46		4.33		6.95	

Table 3. **Nerve conduction data.** pre=pre-treatment; post=post-treatment; R= Right; L= Left; SENS NCS= Sensory Nerve Conduction Studies; MOT NCS= Motor Nerve Conduction Studies; Lat= Latency; AMP= Amplitude; NCV= Nerve Conduction Velocity; NR= No Response; in bold =abnormal values

D	AGE	SIDE	SENS NCS (Sural Nerve)						MOT NCS (Peroneal Nerve)					
			LAT (ms)		AMP(µV)		NCV (m/s)		LAT (MS)		AMP (µV)		NCV (m/s)	
			pre	post	pre	post	pre	post	pre	post	pre	post	pre	post
1	24	R	2.4	2.65	23.7	23.5	58.3	52.8	3.8	3.65	29.9	25.1	43.1	49.2
		L	2.45	2.75	25.3	19.6	57.1	50.9	3.9	3.3	26.1	24.1	44.6	43.5
2	48	R	2.65	2.9	22	21.7	52.8	49.3	**5.65**		8.2		47.1	
		L	2.7	2.55	20.3	21	51.9	48.3	5.15	5.4	8.9	4.3	54.5	49.6
3	35	R	2.6	2.65	11.4	**6.6**	53.8	48.3	5.75	3.65	6.2	7.4	42	38
		L	3.1	2.95	**9**	**6**	45.2	44.5	4.85		7.3		41.8	
4	44	R	2.5	2.35	**8**	**4.4**	56	62.2	3.8	4.2	6.4	3.8	44	42
		L	2.4	2.35	**9.8**	**11.3**	58.3	59.6	4.9		2.6		43.7	
5	45	R	2.8	2.3	**9.9**	17.7	51.8	51.2	3.55	4.3	4.2	1.1	42.4	40.7
		L	2.7	2.35	**10**	19.9	53.8	50.1		4.15		1.9		42.2
6	37	R	2.85	2.65	14.6	14.3	49.1	47.1	4.55	4.75	2.2	8.2	45.9	43.7
		L	2.5		22.9		56							
7	50	R	2.85	2.55	15.3	15.3	**41.5**	**42**	4.55	4.85	8.5	6	**33.3**	**32.9**
		L	2.5	2.4	11.7	15.9	48	46.2		4.85		6		**33.1**
8	41	R	2.75	3.35	**9.3**	**8.2**	54.5	41.8	3.95	4.75	5.3	7.1	41.9	38.8
		L	2.9	3.3	**10.3**	**8.1**	50	42.4		4.9		4.1		37.5
9	28	R	2.3	2.2	15.7	15	60.9	63.6	4.95	3.35	5.5	7.9	43.1	45.6
		L	2.6		9.9		53.7							
10	38	R	2.75		17.6		50.9		6.3	5.3	10.6	5	42.2	43.5
		L	2.15	2.6	21.4	14.7	60.5	53.8						
11	37	R	2.35	2.85	**9.8**	**11.1**	59.6	49.1	4.55	4.45	7.8	3.9	43.8	44.6
		L	2.45		**7.3**		57.1							
12	64	R	**NR**	**NR**	**NR**	**NR**	**NR**	**NR**	4.2	4.8	**1.2**	**1.3**	48.8	48
		L	**NR**	**NR**	**NR**	**NR**	**NR**	**NR**						
13	31	R	2.75	2.95	15.5	20.8	50.9	47.5	**5.85**	**6.25**	2.4	3.5	24.3	**22.5**
		L	2.55	3	19.3	24.8	54.9	46.7	**6.75**	**6.5**	2.3	3.4	25.2	**24.6**

NORMATIVE VALUES

	Amp	Lat	NCV
**Peroneal Nerve**			
Upper limit (mean + 2SD) Range 23-35 y-o	5.4 +/- 3	3.7 +/- 1.8	49·5 +/- 10·8
Upper limit (mean + 2SD) Range 44-65 y-o	5.0 +/- 2.6	3.7 +/- 1.4	48·3 +/-9·2
**Sural nerve**			
Upper limit (mean + 1SD) Range 23-35 y-o	20.9 +/- 8.0	2.7 +/- 0.3	52·5 +/- 5·6
Upper limit (mean + 1SD) Range 44-65 y-o	17.2 +/- 6.7	2.8 +/- 0.3	51·1 +/- 1·3
**Tibial Nerve**			
Upper limit (mean + 2SD)	5.8 +/- 3.8	3.96 +/- 2.00	48.5 +/- 7.2
**Superfical Peroneal Nerve**			
Upper limit (mean + 1SD) Age over 15 y-o	13.9 +/-4.6	2.24 +/- 0.49	47.3 +/- 3.4

Table 4. Spectroscopic and Diffusion Tensor Imaging data

ID	AGE	Spectroscopy								Difusion Tensor Imaging	
		Myo/Cr		NAA/Cho		NAA/Cr		Cho/Cr		FA	
		PO		PO		PO		PO		CST	
		pre	post	pre	post	pre	post	pre	post	pre	post
1	24	**0.8838**	**0.9046**	2.10	2.65	**1.6879**	**1.3367**	0.8041	0.5043	0.515269	**0.494690**
2	48	**0.8387**	0.7861	2.32	2.41	1.7097	**1.3668**	0.7359	0.5661	0.529420	0.528411
3	35	**1.0382**	**0.8109**	**1.31**	1.74	**1.6279**	**1.6218**	**1.2452**	**0.9345**	0.554577	0.545326
4	44	0.7874	**0.8153**	**3.54**	1.73	**1.6556**	1.8241	0.4677	**1.0514**	0.512509	0.479226
5	45	**0.9500**	**0.8596**	1.52	1.89	2.0600	**1.2682**	**1.3596**	0.6696	0.522925	0.513929
6	37	**0.9652**	**1.1509**	1.76	1.45	**1.4299**	**0.9899**	0.8133	0.6818	**0.497866**	**0.4635**
7	50	**0.9858**	**0.9693**	2.16	**1.20**	**1.4871**	**1.4559**	0.6897	**1.2145**	**0.492247**	**0.477074**
8	41	0.7809	0.7233	1.65	2.18	**1.5551**	**1.3841**	**0.9416**	0.6360	0.530123	0.526146
9	28	0.7474	**0.8401**	**6.79**	2.37	**1.6173**	**1.6132**	**0.2382**	0.6808	0.518413	0.512709
10	38	**0.8376**	**1.0453**	1.73	**3.58**	1.8619	**1.4057**	**1.0754**	**0.3928**	**0.485787**	**0.491679**
11	37	0.7669	**1.2308**	2.68	**4.66**	**1.6889**	**1.0735**	0.6296	**0.2306**	0.554720	0.534809
12	64	**1.0243**	**0.9385**	**3.25**	**3.14**	**1.5255**	**1.3483**	0.4699	0.4298	0.531937	0.506943
13	31	**0.9693**	**0.9111**	**1.20**	1.70	**1.4559**	**1.5715**	**1.2145**	**0.9258**	not done	not done
mean		0·8904	0.9220	2.4614	2.3611	1.6433	1.4046	0.8219	0.6860	0.5205	0.5062
SD		0·10	0.15	1.48	0.96	0.17	0.22	0.34	0.28	0.02	0.03
Normative values mean		0·5700		2.2000		1.4400		0.6600		0.5700	
(mean +/- 3 SD)		< 0·81		2.95-1.45		> 1.800		0.42-0.90		> 0.51	

FA=Fractional anisotropy; CST= Corticospinal Tract; PO=Parieto-occipital white matter; in bold: abnormal values; in red: withdrawn from treatment; Myo: myo-inositol; Cre: creatine; Cho: choline; NAA: N-acetylaspartate

Normative values (spectroscopie): Garnett et al; Brain 2000;123 (Pt 7):1403-1409.
Normative values (DTI): Dubey et al; Ann Neurol 2005;58:758-766.
